# Feasibility of controlling hookworm infection through preventive chemotherapy: a simulation study using the individual-based WORMSIM modelling framework

**DOI:** 10.1186/s13071-015-1151-4

**Published:** 2015-10-22

**Authors:** Luc E. Coffeng, Roel Bakker, Antonio Montresor, Sake J. de Vlas

**Affiliations:** Department of Public Health, Erasmus MC, University Medical Center Rotterdam, P.O. Box 2040, 3000 CA Rotterdam, The Netherlands; Department of Control of Neglected Tropical Diseases (NTD), World Heath Organization, Avenue Appia 20, 1211 Geneva, Switzerland

**Keywords:** Hookworm, Mass drug administration, Feasibility of control, Systematic non-participation, Individual-based modelling

## Abstract

**Background:**

Globally, hookworms infect 440 million people in developing countries. Especially children and women of childbearing age are at risk of developing anaemia as a result of infection. To control hookworm infection and disease (i.e. reduce the prevalence of medium and heavy infection to <1 %), the World Health Organization has set the target to provide annual or semi-annual preventive chemotherapy (PC) with albendazole (ALB) or mebendazole (MEB) to at least 75 % of all children and women of childbearing age in endemic areas by 2020. Here, we predict the feasibility of achieving <1 % prevalence of medium and heavy infection, based on simulations with an individual-based model.

**Methods:**

We developed WORMSIM, a new generalized individual-based modelling framework for transmission and control of helminths, and quantified it for hookworm transmission based on published data. We simulated the impact of standard and more intense PC strategies on trends in hookworm infection, and explored the potential additional impact of interventions that improve access to water, sanitation, and hygiene (WASH). The individual-based framework allowed us to take account of inter-individual heterogeneities in exposure and contribution to transmission of infection, as well as in participation in successive PC rounds.

**Results:**

We predict that in low and medium endemic areas, current PC strategies (including targeting of WCBA) will achieve control of hookworm infection (i.e. the parasitological target) within 2 years. In highly endemic areas, control can be achieved with semi-annual PC with ALB at 90 % coverage, combined with interventions that reduce host contributions to the environmental reservoir of infection by 50 %. More intense PC strategies (high frequency and coverage) can help speed up control of hookworm infection, and may be necessary in some extremely highly endemic settings, but are not a panacea against systematic non-participation to PC.

**Conclusions:**

Control of hookworm infection by 2020 is feasible with current PC strategies (including targeting of WCBA). In highly endemic areas, PC should be combined with health education and/or WASH interventions.

**Electronic supplementary material:**

The online version of this article (doi:10.1186/s13071-015-1151-4) contains supplementary material, which is available to authorized users.

## Background

Globally, over 1 billion people in developing countries are infected with soil-transmitted helminths (STH), of which about 440 million people are infected with at least one type of hookworm (*Necator americanus* or *Ancylostoma duodenale*) [1]. Adult hookworms attach themselves to the intestinal mucosae to feed on host blood, causing leakage of intestinal blood and thus contributing to the development of iron-deficiency anaemia. The risk of anaemia is highest in heavily infected individuals, as well as children and women of childbearing age (WCBA), given their naturally low iron reserves [2, 3]. To control the global disease burden of hookworm and other STH infections, the World Health Organization (WHO) has set the operational target to provide regular preventive chemotherapy (PC) to at least 75 % of the population at highest risk for hookworm morbidity by 2020, i.e. pre-school (preSAC) and school-age children (SAC), and WCBA. The associated parasitological goal is to reduce the prevalence of medium and heavy infection (≥2000 eggs per gram (epg) faeces) to levels under 1 % among preSAC, SAC, and WCBA by 2020 [4]. PC targeting preSAC and SAC is typically implemented at the level of schools, its frequency (annual or semi-annual) depending on pre-control STH infection levels [5]. PC targeting WCBA is not yet widely implemented, but planned to be rolled out over the coming years. Mass drug administration (MDA; i.e. PC targeting the whole population) against STH is not officially implemented, but practically taking place in areas where The Global Program for Elimination of Lymphatic Filariasis [6] is fighting lymphatic filariasis by means of MDA using a combination of albendazole and diethylcarbamazine or ivermectin. Similarly, mass drug administration with ivermectin by the African Programme for Onchocerciasis Control will most likely have already had a significant impact on the STH burden [7].

Hookworm epidemiology differs from that of other STH, bringing with it a particular challenge for control. The intensity and prevalence of hookworm infection typically rise during childhood and reach a plateau in adult persons, whereas in ascariasis and trichuriasis, infection levels typically peak in children and then decline with age [2, 3]. An explanation for this difference is that ascariasis and trichuriasis are transmitted through ingestion of worm eggs, a mechanism that is strongly related to hygienic practices, which are typically poorer in children than in adults. In contrast, hookworm infection is acquired through larval skin penetration, a mechanism that is related to footwear practices and movement patterns, which are relatively stable over different ages. Further, although not practiced in all endemic regions, the use of human excrements as fertiliser (*night soil*) provides an additional mechanism by which adults are exposed to hookworm infection. Given that current PC programs mostly target preSAC and SAC, while adult hosts harbour most hookworms and therefore probably contribute most to transmission, the feasibility of controlling hookworm infection by 2020 with current PC strategies can be questioned [8].

Here, we predict the feasibility of achieving <1 % prevalence of medium and heavy hookworm infection by 2020 with the currently recommended PC strategies (annual or semi-annual PC at 75 % coverage, targeting preSAC, SAC, and WCBA). We further predict the impact of more intense PC strategies (higher frequency and coverage) and targeting of the entire population of age two and above. We performed simulations in WORMSIM, a newly developed generalised modelling framework for transmission and control of helminth infections. The individual-based nature of WORMSIM allows us to take account of important sources of heterogeneity at the individual level, notably exposure and contribution to transmission of infection, as well as participation in successive PC rounds. We evaluated the feasibility of control for various scenarios pertaining to pre-control infection levels and patterns in individual participation in PC, including varying levels of systematic (non-)participation of a subgroup of individuals.

## Methods

### General outline of the WORMSIM modelling framework

**Fig. 1:**
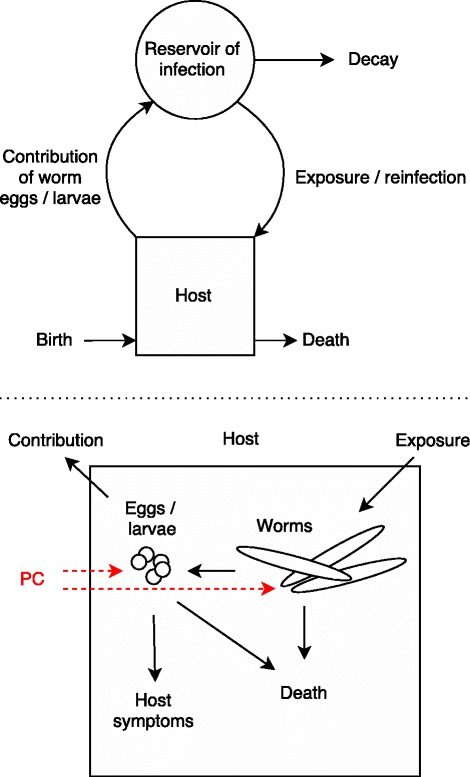
Schematic representation of the WORMSIM modelling framework structure. In the model, the life histories of multiple hosts and worms within hosts is simulated. Preventive chemotherapy (PC) is assumed to affect survival and/or reproductive capacity of worms and their offspring. The reservoir of infection can be taken to represent either a cloud of vectors (as in transmission of filariases), or an environmental reservoir of infection (as in transmission of soil-transmitted helminths)

WORMSIM is a generalised individual-based modelling framework for transmission and control of helminth infections in humans (Fig. 1), and is based on earlier individual-based models for onchocerciasis, schistosomiasis, and lymphatic filariasis [9–11]. Here we describe the general outline of the framework, partly based on an earlier description of ONCHOSIM [12]. Additional file 1 provides more technical details and the mathematical formulae underlying the model. Additional file 2 contains a zip-compressed version of the WORMSIM modelling framework, including example input files.

#### Human demography

WORMSIM simulates the life histories of individual humans and individual worms within humans. Simulated humans are born and die in a stochastic fashion, based on user-specified fertility and life tables. The size of the simulated human population depends on the specified fertility and survival rates, as well as an optionally defined maximum population size. When the maximum population size is exceeded, a random fraction of the population is removed from the simulation. Other than that, WORMSIM simulates a closed population, meaning that there is no migration of humans into or out of the population.

#### Acquisition of infection and worm reproduction

Simulated humans are exposed and contribute to a central reservoir of infection, which can be taken to represent a cloud of vectors that transmit infection (e.g. as for onchocerciasis), or an environmental reservoir of infective material (more details below). The rate at which each simulated individual is exposed to the reservoir of infection may depend on the season of the year, age, sex, and random personal factors defined in terms of relative individual exposure (following some distribution with mean one, for which we here use a gamma distribution with equal shape and rate *α*_*Exi*_). The overall exposure rate in the simulation (i.e. for a person with relative exposure 1.0) is defined in terms of parameter *ζ*. When infective material from the reservoir is successfully transmitted to an individual, it may develop into a male or female adult worm. Once a female worm lives beyond a user-specified prepatent period, she starts producing eggs or larvae, as long as she is regularly inseminated by a patent male worm present in the same host. The egg or larval production can be specified to depend on the age of the female worm. The lifespan of adult worms is simulated in a stochastic fashion, given some user-defined, positively bounded continuous distribution.

#### Transmission of infection to environmental reservoir or vector

Humans containing reproductive adult worms contribute infective material (larvae or eggs) to the central reservoir of infection. An individual’s contribution rate may depend on the season of the year, age, sex, and random personal factors. When the reservoir is taken to represent a cloud of vectors that transmit larvae (as for onchocerciasis), infective material passes through the reservoir instantaneously (a reasonable assumption when passage through the vector is short relative to the discrete simulation time steps of 1 month), such that the force of infection acting on the human population is always proportional to the amount of infective material contributed to the central reservoir by the human population. When the reservoir is taken to represent an environmental reservoir of infection (as for soil-transmitted helminths), infective material is considered to accumulate and decay within the reservoir, given the total contribution of the human population and some exponential decay rate for infective material in the reservoir. In this case, the force of infection acting on the human population is proportional to the amount of infective material currently present in the environmental reservoir.

#### Density dependence in transmission

Density dependence in transmission can be specified at several points in the transmission cycle: uptake of infectious material by the central reservoir of infection (e.g. due to limited vectorial capacity), worm fecundity (e.g. due to host immune response and/or competition for nutrients), and worm establishment (e.g. worms already present in the host may trigger partial immunity).

#### Drug treatment

Drug treatment can be specified to temporarily and/or permanently reduce the reproductive capacity of female worms, and to kill adult worms and/or infective material (larvae or eggs) present in the host. The probability that an individual participates in a PC programme is determined by the overall PC coverage level, and the relative probability of participating, given an individual’s age, sex (taking into account treatment eligibility), and/or a lifelong compliance factor. Individual participation to PC is assumed to be either random (given age and sex), fully systematic (given the lifelong compliance factor), or a mix of random and systematic participation (see Additional file 1 for details). In addition, the user can specify that treatment fails in a random fraction of people (e.g. due to malabsorption).

### WORMSIM quantification for hookworm transmission

In Additional file 1, we provide an overview of the quantification of WORMSIM and underlying assumption in Additional file 1: Table A1–3. Below, we provide an overview.

#### Adult parasites

We set the average lifespan of hookworms in the human host to 3 years [3, 13–15], and assumed that the worm mortality rate increases linearly with worm age (i.e. worm lifespan follows a Weibull distribution with mean 3.0 and shape 2.0). Based on literature, we assumed that hookworms can reproduce after a fixed prepatent period of 7 weeks [2, 3, 13, 16]. Fecundity-related parameters were set such that egg production was independent of female worm age, and female worms could produce eggs as long as at least one male worm was also present in the host. The total egg output of all female worms in a host was assumed to be negatively density-dependent on the total number of female worms *x*. This density dependence was defined in terms of the hyperbolic saturating function *αx*/(1 + *αx*/*β*), where *α* is the average egg production per female worm in absence of density dependence (*α* = 200 epg [17]), and *β* is the average maximum egg output for a host (see section Endemicity scenarios below for details about quantification of *β*).

#### Host suitability for infection

To capture inter-individual variation in host suitability for worm infection (e.g. due to genetic factors [18–20], nutrition status, and/or immunocompetence), each individual’s saturation level for total egg output *β*_*i*_ was assumed to be a random lifelong trait with inter-individual variation proportional to a gamma distribution with mean 1.0. As there simply is no field data on this particular host characteristic, we first assumed that variation in relative host susceptibility was low with 95 %-CI: 0.74–1.30 (gamma distribution with shape and rate equal to 50), such that predictions were very similar to the assumption of “no variation in susceptibility” (i.e. relative susceptibility = 1.0, which has been typically employed so far). Next, we repeated the whole analysis with an arbitrary, much more extreme assumption about high variation in host susceptibility (95 %-CI: 0.12–2.29; shape and rate equal to two, such that the value of zero relative susceptibility still has zero density).

#### Larvae in the environment

**Fig. 2:**
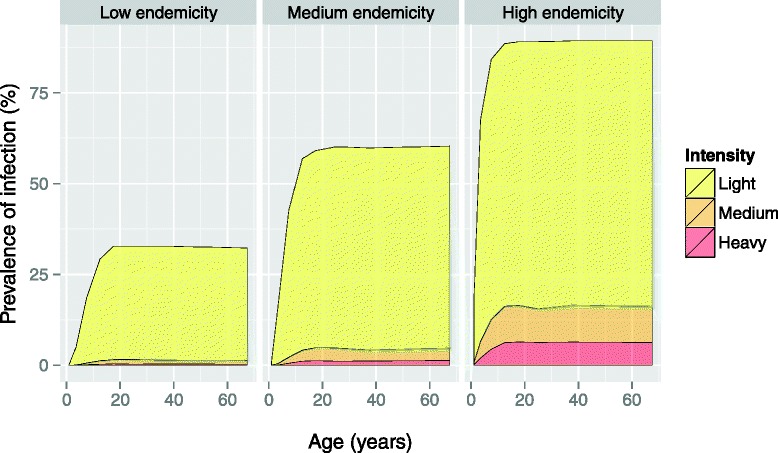
Pre-control distribution of infection intensity (stacked), as predicted by WORMSIM for three endemicity scenarios. The age-pattern is driven by the assumption that host exposure increases linearly from zero to one between ages zero and ten, and is stable from then onwards, resulting in the typical plateau in infection levels from about age 20 onwards [16]. Simulated egg counts are based on single Kato-Katz slides of 41.7 mg with negative binomial sampling error (*k*_Kato-Katz_ = 0.40, based on an analysis of field data [18])

The average lifespan of larvae in the environmental reservoir was set to 2 weeks, assuming an exponential distribution [2, 16, 21]. Because the survival of larvae may vary geographically with environmental conditions (average humidity and temperature), we alternatively assumed that average larval lifespan is 4 weeks (implying higher reinfection rates between PC rounds). Host contribution and exposure to the environmental reservoir were assumed to be perennial, with age-patterns increasing linearly from zero to one between ages zero and ten, such that we achieve the typical age pattern of infection levels increasing with age until they reach a plateau at age 20 (Fig. 2) [16]. In addition, we assumed that individual exposure and contribution rates vary randomly due to personal factors (for quantification, see section Endemicity scenarios). Contribution and exposure were assumed to be perfectly correlated for each individual.

#### Effect of preventive chemotherapy

We assumed that treatment with albendazole (ALB) or mebendazole (MEB) kills a given fraction of prepatent and adult worms, and has no effect on worm fecundity. We further assumed that the proportion of worms killed by ALB or MEB is equal to observed reductions in mean egg counts (95 and 80 % respectively) [22]. This is a reasonable assumption, as in the cited study reductions in hookworm egg counts were not correlated with pre-treatment egg counts. Drug efficacy was assumed to be equal for all host and worm ages.

#### Parasitological diagnosis

Simulation output on infection levels was defined in terms of prevalence of no, light, medium, and heavy infection (cut-offs: 1, 2000, 4000 epg), based on single Kato-Katz slides of 41.7 mg. Kato-Katz slides are wet mounts of faecal samples, which are systematically examined under a microscope to count worm eggs [23]. The sensitivity of Kato-Katz slides to detect infection increases with the number of eggs in the sample. To simulate this, we assumed that sampling error in Kato-katz slides follows a negative binomial distribution with mean egg count as predicted by WORMSIM for a given person, and aggregation parameter *k*_Kato-Katz_ = 0.40, based on an analysis of repeated slides from 2083 Ugandan individuals (see Additional file 3). We assumed perfect specificity of Kato-Katz testing (i.e. as if performed by a trained and experienced laboratory technician). Simulation output on prevalence of infection was stratified for the following sub-populations: *infants* (age <2), *pre-SAC* (age 2–5), *SAC* (age 5–15), *WCBA* (females of age 15–45), and *other* (females of age 45 and above, and males of age 15 and above).

### Simulations

#### Comparison to field data

First, we compared WORMSIM predictions for the impact of PC to field trial data on trends in hookworm burden in a population of Vietnamese WCBA who were offered 4-monthly treatment with albendazole for 1 year, and 6-monthly from then onwards [24, 25]. For this setting, we assumed that the average saturation level for egg output was 1500 epg (between 1113 and 1943 for 95 % of individuals) or 2000 epg (1484–2591), and that the coverage of mass treatment targeting WCBA was as reported during a single cross-sectional survey (85.8 %) and remained stable for the entire 54-month study period. Because we had no exact information on the timing and coverage of PC targeting preSAC and SAC in the study area, we assumed that these were treated at the same time as the WCBA, and at equal coverage.

#### Endemicity scenarios

**Fig. 3:**
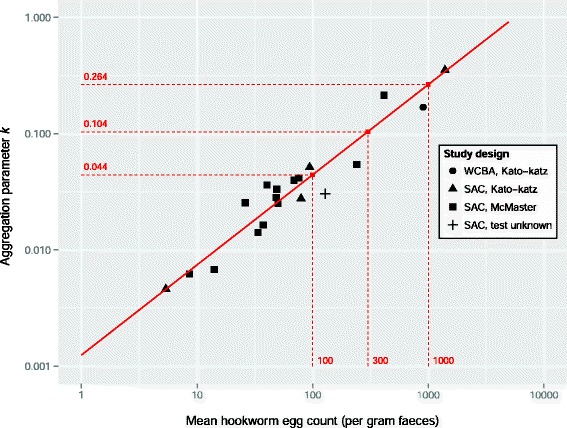
Association between mean hookworm egg count and aggregation parameter *k* estimated from published studies. The legend indicates underlying study design in terms of study population (WCBA = women of child bearing age; SAC = school-age children) and the parasitological test used [22, 26–31]. Taken together, the literature studies covered 24,758 individuals, for each of which the intensity of infection was known in terms of no, light, medium, or heavy infection (cut-offs: 1, 2000, and 4000 epg). The solid red line represents the linear association between the logarithms of the mean egg count and aggregation parameter *k*, taking account of uncertainty in both quantities (*ρ* = 0.92, 95 %-Bayesian credible interval 0.78–0.98). *Red dashed lines* represent the three pre-control endemicity levels for which simulations were performed in WORMSIM

For the purpose of predicting the impact of PC, we defined three endemicity levels (high, medium, low) representative of field conditions (Fig. 3), based on an analysis of literature data [22, 26–31] (previously collated and described elsewhere [32]). Each endemicity level was defined in terms of mean egg counts and a negative binomial aggregation *k*, from which we derived target values for the distribution of intensity of infection (none, light, medium, heavy) to reproduce in WORMSIM (Table 1). The endemicity scenarios were arbitrarily chosen such that each scenario had a pre-control prevalence of medium and heavy infection (≥2000 epg) in 10–14 year old children of at least 1 % (such that preventive chemotherapy is still indicated), and such that together, the endemicity scenarios spanned the range of infection levels observed in literature (Fig. 3). For technical details on how these endemicity scenarios were arrived at, see Additional file 3.

**Table 1:** Quantification of endemicity scenarios, based on the association between mean and aggregation of egg counts

	Scenario quantification derived from statistical model^a^		Pre-control prevalence of infection in school-age children (age 10–15)			
Endemicity scenario	Mean egg count (epg)	Aggregation parameter (*k*)	Light infection (%)	Medium infection (%)	Heavy infection (%)	Any infection (%)
High	1000	0.264	73.2	8.8	6.7	88.6
Medium	300	0.104	52.1	2.8	1.4	56.3
Low	100	0.044	27.6	0.9	0.3	28.8

^a^See also Fig. 3 and Additional file 3

The average saturation level for host egg output was set to *β* = 1500 epg such that WORMSIM could reproduce the distribution of intensity of infection for the highly endemic scenario, and such that it could also still simulate sustained transmission for the low endemicity scenario. We also investigated the alternative assumption that average saturation level in egg output is higher (*β* = 2000 epg, i.e. weaker density dependence in transmission). Similarly, for low and medium endemic areas, we investigated the alternative assumption that average saturation level is lower (*β* = 1000 epg, i.e. stronger density dependence). Next, we performed a grid search to quantify the remaining free transmission parameters *ζ* and *α*_*Exi*_ in WORMSIM, so as to reproduce the distribution of light, medium, and heavy infection as expected for each endemicity level (see in Additional file 3: Table A3–2).

#### Preventive chemotherapy programmes

Next, for each endemicity scenario (high, medium, low), we performed 250 repeated simulations to predict the average impact of PC on hookworm transmission in a closed population of about 400 individuals, given the estimated efficacy of a single drug treatment. Simulations that resulted in interruption of transmission before start of control were dropped from the analyses (this occurred in about 40 % of the simulations for the low endemic scenario with *β* = 2000). We varied assumptions about PC frequency (annual, semi-annual, 4-monthly, or quarterly), target population (preSAC and SAC; preSAC, SAC and WCBA; or total population of age two and higher), coverage of target population (75 %, the WHO operational nation-level target, or 90 %, the level of coverage typically achieved in individual schools), and patterns in participation (random, mixed, or systematic, see in Additional file 1: Figure A1-2 for illustration) as also previously used in mathematical modelling of onchocerciasis [9]. We assumed that PC coverage is stable over time, once PC is implemented, even though national coverage rates for preSAC and SAC published by WHO increase over time [33]. However, these national figures are based on a mix of local coverage rates of mostly zeroes (no PC) and high rates (75–95 % wherever PC is successfully implemented), and therefore mainly reflect geographical scaling up of PC. Because transmission takes place locally, we chose to simulate stable coverage only in our main analysis. Only to compare our model predictions with those by Truscott et al. [34], who assume that local PC coverage levels do follow the national average trend, we also provide predictions for this scenario (i.e. assuming a linear increase in coverage of preSAC and SAC, starting at 0 % in 2002, and increasing up to 75 % in 2020, and stable from then onwards).

#### Health education and WASH

To explore the potential effects of health education and improved access to water, sanitation, and hygiene (WASH), we simulated the impact of a 50 % reduction in contribution of all individuals to the environmental reservoir (WORMSIM does not yet support an effect of WASH on exposure, e.g. as a result of improved flooring in dwellings).

## Results

### Comparison of prediction to field data

**Fig. 4:**
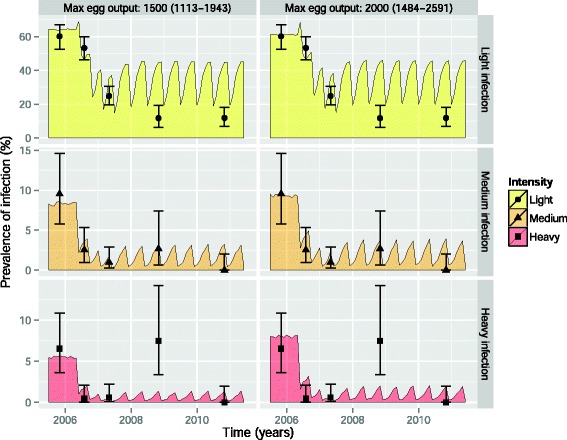
Comparison of WORMSIM predictions to longitudinal data on impact of mass drug treatment with albendazole. The data [24] consist of the number of women of childbearing age with no, light, medium, and heavy infection (cut-offs: 1, 2000, 4000 epg) based on a single Kato-Katz slide, determined at five time points: pre-control (October 2005), and 3, 12, 30, and 54 months after the first round of PC. Data were collected by means of cross-sectional surveys, i.e. not the same women were necessarily sampled at each time point. WORMSIM parameters for the overall transmission rate (ζ) and exposure heterogeneity (*α*_*Exi*_) were tuned to reproduce pre-control distribution of intensity of infection, assuming that the average saturation level for host egg output is 1500 or 2000 epg. Based on published data, we simulated 4-monthly treatment with albendazole targeting WCBA for 1 year, and 6-monthly from then onwards [24, 25]. Mass treatment coverage was assumed to be as reported during a single cross-sectional survey (85.8 %) and was assumed to remain stable for the entire 54-month study period. Because we had no exact information on the timing and coverage of PC targeting preSAC and SAC in the study area, we assumed that these were treated at the same time as the WCBA, and at equal coverage. Error bars represent 95 %-Bayesian credible intervals

WORMSIM-predicted trends for light, medium, and heavy infection were in good agreement with field data on WCBA from Vietnam at 3 and 12 months after the first treatment rounds (Fig. 4). However, at 30 months after start of the PC programme, the reported prevalence of heavy infection was higher than predicted by WORMSIM. At 54 months, the reported prevalence of light and medium infection was lower than predicted by WORMSIM. These differences may be explained by sampling issues at 30 months and changes in behaviour over time (see Discussion).

### Predicted impact of preventive chemotherapy in low and medium endemic areas

**Fig. 5:**
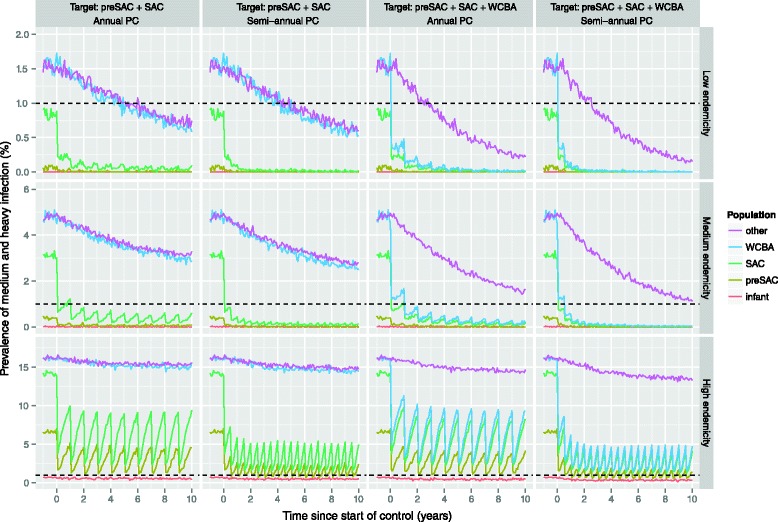
Impact of targeted preventive chemotherapy with albendazole, as predicted by WORMSIM. The horizontal dashed black line indicates the WHO target level of 1 % prevalence of medium and heavy infection. Coloured graph lines in each panel represent predicted trends in prevalence of medium and heavy infection in different subpopulations (see legend on the right). Each row of panels represent one of three pre-control endemicity levels, while columns represent different preventive chemotherapy (PC) strategies: annual vs. semi-annual and targeting of only pre-school (preSAC) and school-age children (SAC) vs. aforementioned plus women of child bearing age (WCBA). PC coverage is assumed to be 75 %, in line with the WHO operational target, and individual participation in PC is determined by a mix of random and systematic factors

For the low and medium endemic scenarios, current PC strategies targeting only children (annual or semi-annual PC at 75 % coverage) are predicted to achieve control of hookworm infection (prevalence of medium and heavy infection <1 %) in preSAC and SAC within a few years (Fig. 5, first two columns). However, PC specifically also targeting WCBA (third and fourth column) is required to also achieve control in WCBA (blue line) within the same time span (low endemic scenario), or at all (medium endemic scenario). For women over 45 years of age and men over 15 years of age (pink line; not targeted by PC) PC targeting preSAC, SAC, and WCBA provides some benefit through indirect transmission effects, but this decreases with higher pre-control infection levels. These findings are independent of the choice of drug: ALB (Fig. 5) or MEB (Additional file 4).

### Predicted impact of preventive chemotherapy in highly endemic areas

**Fig. 6:**
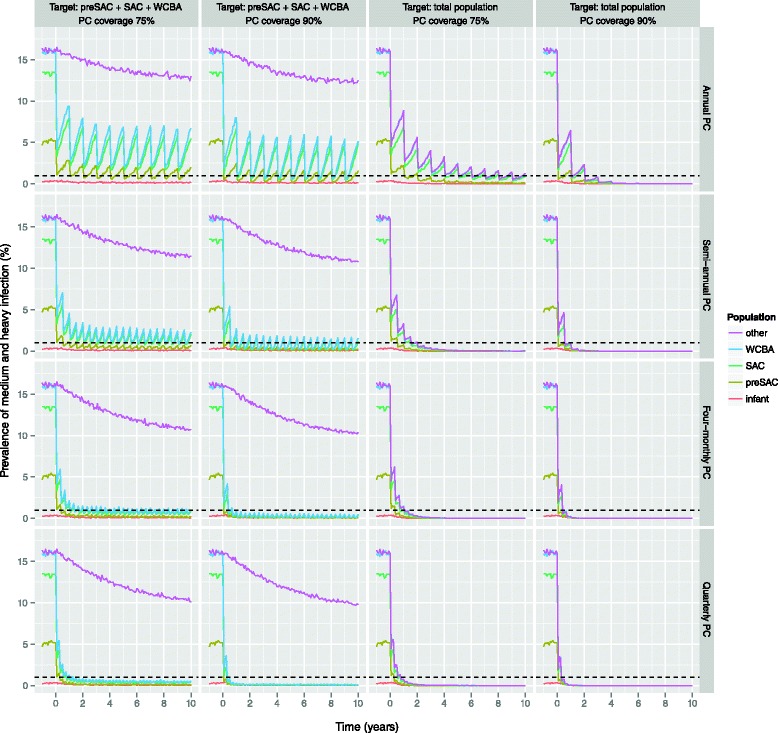
Impact of more intensive preventive chemotherapy with albendazole in highly endemic areas, as predicted by WORMSIM. All panels pertain to the highly endemic scenario. The horizontal dashed black indicates the WHO target level of 1 % prevalence of medium and heavy infection. Panels from left to right represent different PC target populations (preSAC, SAC, and WCBA vs. total population of age two and above), and PC coverage (75 % vs. 90 %). Panels from *top* to *bottom* represent PC implemented at different frequencies (annual vs. semi-annual vs. 4-monthly vs. quarterly PC)

**Fig. 7:**
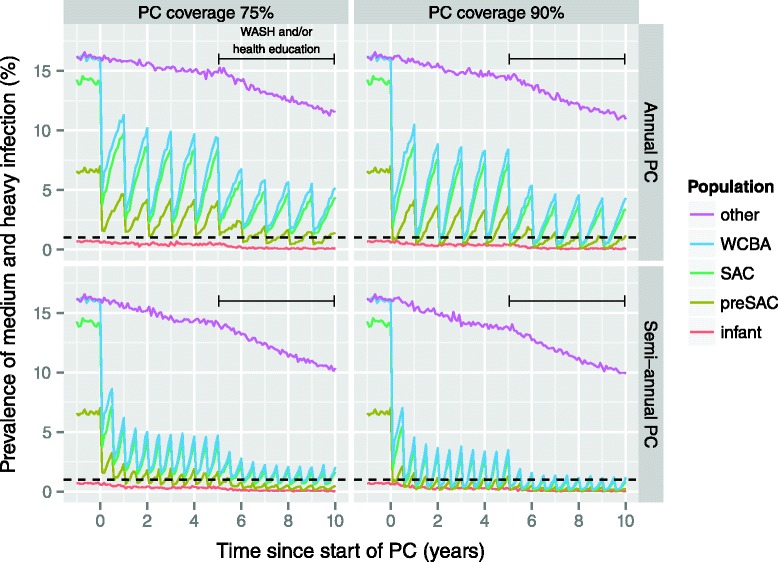
Impact of preventive chemotherapy and WASH and/or health education on hookworm infection levels. All panels pertain to the highly endemic scenario with PC targeted at pre-school and school-age children, and women of childbearing age. PC is assumed to start at time point 0, whereas WASH interventions are assumed to be implemented and effective from 5 years later onward (horizontal solid black line). The horizontal dashed black indicates the WHO target level of 1 % prevalence of medium and heavy infection. We assumed that WASH reduces the contribution of all individuals to the environmental reservoir by 50 % (WORMSIM does not yet support an effect of WASH on exposure of hosts, e.g. as a result of improved flooring in dwellings). Panels from *left* to *right* represent different levels of PC coverage (75 % vs. 90 %). Panels from top to bottom represent PC strategies at different frequencies (annual vs. semi-annual). Individual participation in PC is determined by a mix of random and systematic factors

For the highly endemic scenario, semi-annual PC at 75 % coverage is not sufficient to achieve control (Fig. 5). However, we predict that control in preSAC, SAC, and WCBA can be achieved in highly endemic areas by implementing PC with ALB either quarterly at 75 % coverage, 4-monthly at 90 % coverage, or semi-annually targeting the whole population (under our standard assumption of density-dependent fecundity with *β* = 1500; Fig. 6). In case PC with MEB is implemented, more intensive strategies are required (90 % coverage and/or higher frequency; Additional file 1). Alternatively, control of hookworm infection in highly endemic settings may also be achieved by a combination of the standard strategy of semi-annual PC at 90 % coverage and interventions that reduce the contribution of infectious material by each host by 50 % (Fig. 7). However, under the alternative assumption of lower density dependence in worm fecundity (*β* = 2000), we predict that control can even be achieved with semi-annual PC with ALB alone, when implemented at 90 % coverage and targeting preSAC, SAC, and WCBA.

### Impact of systematic (non-)participation

**Fig. 8:**
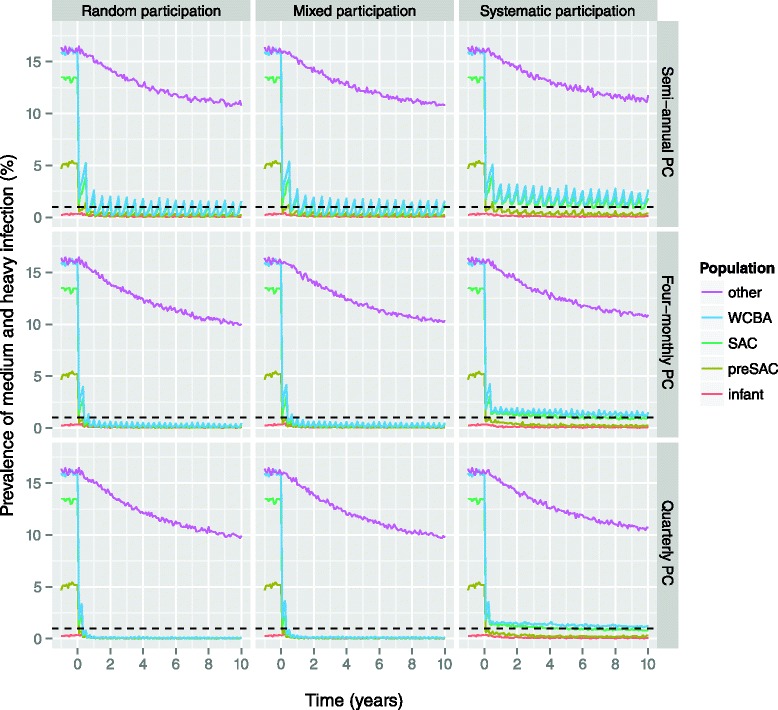
Effect of systematic (non-)participation on impact of preventive chemotherapy with albendazole, as predicted by WORMSIM. All panels pertain to the highly endemic scenario with PC targeted at pre-school and school-age children, and women of childbearing age, implemented at 90 % coverage. The horizontal dashed black indicates the WHO target level of 1 % prevalence of medium and heavy infection. Panels from left to right represent different patterns in individual participation to PC. Random participation (*left column*) means that eligible individuals participate completely at random; systematic participation (*right column*) means that an individual either always participates (if eligible) or never; in the mixed participation pattern (*middle column*), some individuals are systematically more likely to participate than others (but everyone will participate at some point). Panels from top to bottom represent PC implemented at different frequencies (semi-annual vs. 4-monthly vs. quarterly PC)

Figure 8 illustrates how systematic (non-)participation of individuals to PC with albendazole can dramatically decrease the impact of PC, regardless of treatment frequency. However, as long as all individuals have a chance to be treated at some point (mixed participation), the impact of PC was predicted to be very similar to that in the scenario of completely random participation. This finding was the same for PC with MEB (Additional file 4).

All aforementioned findings with regard to achieving control were robust to alternative assumptions about the level of density dependence in transmission, inter-individual variation in host suitability for infection, and the lifespan of larvae in the environmental reservoir (Additional file 4).

### Impact of PC during scaling up

**Fig. 9:**
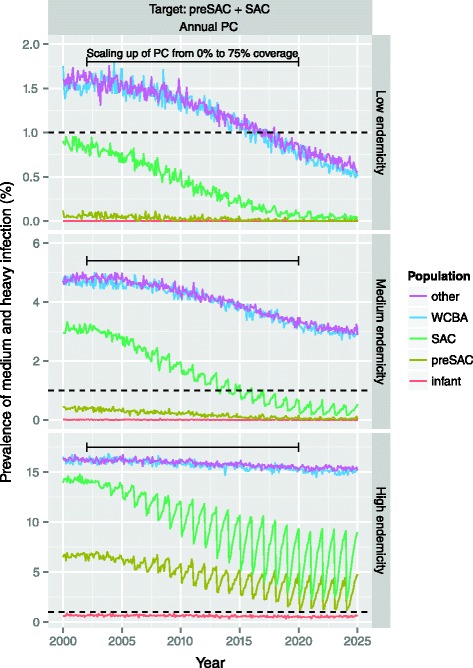
Impact of scaling up annual preventive chemotherapy with albendazole targeting pre-school and school-age children. PC coverage is assumed to increase from 0 % in 2002 to 75 % in 2020 (horizontal solid black line), and to be stable from then onwards. We further assume that individual participation in PC is determined by a mix of random and systematic factors

Figure 9 illustrates the impact of annual PC targeting children, with coverage scaling up from 0 to 75 % between 2002 and 2020, based on trends in national coverage as reported by WHO.

## Discussion

In low and medium endemic settings, achieving control of hookworm infection in preSAC and SAC (i.e. prevalence of medium and heavy infection <1 %) can most likely be achieved with current PC strategies (annual or semi-annual PC at 75 % or 90 % coverage) within as little as 1 to 2 years. Although adults benefit indirectly from PC programs targeting children, this effect is not enough to control of hookworm infection in WCBA in a timely fashion; the latter is best achieved by also actively targeting WCBA. This will be especially important in Asia, where in some localities infection levels keep on rising with age into late adulthood. For low and medium endemic settings, control can be achieved equally well with MEB and ALB.

For highly endemic areas, we predict that control of hookworm infection is feasible by means of semi-annual PC with ALB at 90 % coverage, targeting preSAC, SAC, and WCBA, combined with concomitant interventions that reduce host contributions to the environmental reservoir of infection by, say, about half (e.g. health education or WASH), if implemented successfully. Furthermore, if infection levels in highly endemic areas are (partly) driven by lower density-dependent worm fecundity (e.g. resulting from lower host immunocompetence, such that people actually do not have excessively many worms, but just very productive worms), control may be even achieved with semi-annual PC alone, implemented at 90 % coverage. More intense PC strategies (higher frequency and coverage) can help speed up control of hookworm infection, and may be necessary in some extremely highly endemic settings. Control programs should however keep in mind that more intense PC is not a panacea against systematic non-compliance to PC, which may occur among adults in particular (who are not necessarily treated centrally at school, like children), as observed in mass drug administration programs against lymphatic filariasis [35]. Further, in settings where transmission is seasonal (rather than perennial, as assumed for our predictions), timing of PC right before the transmission season (warm and humid weather) may provide an additional means of maximizing the impact of control efforts.

WORMSIM could reasonably well reproduce trends in light, medium, and heavy infection in Vietnamese WCBA during 1 year of 4-monthly treatment with albendazole. The high prevalence of heavy infection reported at 30 months after the start of the PC programme is most likely caused by issues related to sampling (only 187 WCBA were tested) and suboptimal PC coverage preceding the 30-month survey. Further, our long-term predictions for prevalence of light infection (at 30 and 54 months) were relatively pessimistic compared to reported prevalence figures. The most likely explanation is that the health education component of the field trial helped improve human behaviour related to contribution and/or exposure to the environmental reservoir of infection (nurses and health workers were actively trained and provided with educational material to use during their repeated contacts with the women). Another explanation may be that albendazole not only kills worms, but also somehow cumulatively impairs reproduction of surviving worms, as has been proposed to be an effect of ivermectin on *Onchocerca volvulus* worms [36].

In our exploratory simulations for the potential effects of health education and WASH, we assumed that a hypothetical intervention reduces the host contribution of infective material to the environmental reservoir by 50 %, and equally so for all hosts (WORMSIM does not yet support interventions that impact host exposure). In reality, the effect of health education and WASH interventions most likely varies between individuals due to behavioural factors, and may therefore be relatively lower at the population level than predicted here. However, while we only considered an impact on host contribution to the environmental reservoir of infection, the impact of health education and WASH on exposure to hookworm infection is probably important as well. For instance, natural dwelling floor types (e.g., earth, sand, dung, or mud) greatly contribute to host exposure to infection, and have been estimated to be responsible for as much as 86 % of all hookworm infections [37]. Therefore, we expect that the overall impact of successfully implemented health education and WASH interventions may be even larger than predicted here. Still, some may argue that a 50% reduction is currently too optimistic, given that so far, large-scale community-based WASH trials in India have shown little impact on latrine use and child health [38, 39]. However, a recent meta-analysis did show strong associations between availability of sanitation and STH infections (odds ratio around 0.5), and use of sanitation and hookworm infection (odds ratio 0.6) [40]. Further, a school-based WASH RCT in Kenya did show a reduction in reinfection rate of about 50 % [41]. Also, the Magic Glasses project, a video-based hygiene education program implemented in Chinese schools, was able to reduce incidence of STH infection in children (after albendazole treatment) by 50 % [42]. It may be feasible to achieve similar impacts with WASH in community settings, if implemented successfully, which will most likely depend more on behavioural factors than bricks and mortar [43].

The literature data underlying our predictions are probably most representative of *Necator americanus*, the most prevalent human hookworm species globally, but whose eggs are morphologically indistinguishable from *Ancylostoma duodenale* eggs. Because the egg production rate of *A. duodenale* is believed to be two to three times higher than that of *N. americanus* [3], our model predictions in terms of absolute egg counts may not apply directly to localities where *A. duodenale* is the prominent hookworm species. Still, assuming that density dependence in transmission of *A. duodenale* and *N. americanus* is comparable (in terms of the ratio of eggs per worms and maximum host egg output), our model predictions also apply to *A. duodenale* in a qualitative sense.

Defining the endemicity scenarios for this study, we assumed that in highly endemic areas, heterogeneity in individual exposure and contribution to the environmental reservoir is lower. This clearly resulted from our analysis of literature data on distribution of intensity of infection (Fig. 3), and seems plausible, as in such areas everybody can be thought to walk barefoot and/or defecate in the same area. It may also be that in low endemic areas, density dependence in worm fecundity is stronger because people are less susceptible to heavy infections, e.g. due to higher immunocompetence. However, the finding that at low and medium intensity levels of hookworm infection, faecal egg reduction rates of albendazole and mebendazole are independent of pre-treatment egg counts (in contrast to ascariasis and trichuriasis, for which negative correlations were observed), suggests that at these intensities of infection, density dependence only plays a marginal role [22]. Furthermore, our sensitivity analyses show that our predictions for achieving control of hookworm infection in low and medium endemic areas are robust to alternative assumptions about the mean level of and variation in host suitability for infection.

As previously suggested [8], our findings confirm that school-based deworming may not always be enough to control hookworm infection in population at high risk for morbidity, and that women of childbearing age should also be targeted with PC. Still, the relative importance of human subpopulations of different ages in hookworm transmission is not well known. Anderson et al. explored different assumptions about the relative contribution and exposure of children and adults to the environmental reservoir [8]. Their findings suggest that if children contaminate the environment relatively more often than adults, the impact of PC targeting only children would be relatively larger and also significantly impact infection levels in adults. However, we argue that this is an unlikely scenario, as children’s contributions would have to be higher than adults’ because of their open defecation practices. However, these practices are most likely also correlated with exposure to infection, while typically, observed infection levels are lower in children. In the current study, we therefore assumed that host exposure and contribution to the environmental reservoir of infection are proportional to each other, and that both increase with age (up to the age of 10 years) as a result of open defecation practices. If after all, children do indeed contaminate the environment relatively more often (but are not exposed relatively more often), PC targeting children will have a larger impact on transmission of hookworm infection than predicted here.

A set of previous STH modelling studies focussing on interruption of STH transmission highlight several issues that may also be important for STH control [13–15]. First of all, to reduce hookworm transmission, high PC coverage of adults is more important than for ascariasis transmission due to different age-patterns in infection levels [13, 14]. This is supported by our conclusion that PC should also target women of childbearing age to control hookworm. Second, helminth mating processes and the dynamics of the environmental reservoir play an important role in elimination of STH [15]. In the current study we account for both by explicitly simulating mating events between male and female worms and decay of larvae in the environment. Mating processes are probably less important for control than elimination of infection as in a control situation, relatively many infected individuals will still harbour multiple worms. However, the lifespan of infective material in the environment may weigh into the speed at which control or elimination is achieved. In our simulations, an average larval lifespan of 2 or 4 weeks did not matter much for the speed at which hookworm control is achieved. However, for ascariasis and trichuriasis, the average lifespan of eggs in the environment (which is in the order of months) will probably play a more important role in control and elimination of infection. We will further examine both aspects (PC coverage of age-groups and egg lifespan) in future modelling studies with WORMSIM.

In a similar modelling study, Truscott et al. take a more global perspective on the impact of current PC strategies on ascariasis, trichuriasis, and hookworm, using national trends on PC coverage [34]. Although Truscott et al. predict trends in average worm burdens, their predictions are qualitatively very similar to ours, in that for hookworm, PC targeting children will not benefit adults very much. An important difference in study design however is that Truscott et al. used data national trends in PC coverage, while in our main analysis, we assumed PC coverage is stable over time. We believe our approach is more representative of local situations where STH control is actually taking place and where PC coverage rates are relatively high compared to the national average. When we employed axxproimately the same assumptions about scaling up of PC as Truscott et al. (PC coverage of preSAC and SAC increases linearly from 0 to 75 % between 2002 and 2020), we get very similar results: the impact of PC only becomes really noticeable when coverage levels approach 50 % (between 2010 and 2015). Still, we believe that these predictions do not realistically represent national or global trends in infection levels, but only a local setting (i.e. community-level) in which PC coverage is scaled up.

## Conclusions

We predict that control of hookworm infection in low and medium endemic areas by 2020 is feasible with current PC strategies, which we consider to include targeting of WCBA. Control of hookworm infection in highly endemic areas may be achieved with a minimum of semi-annual PC with ALB at 90 % coverage, combined with health education and/or WASH interventions. More intense PC strategies (high frequency and coverage) may help speed up control of hookworm infection, and may be necessary in some extremely highly endemic settings.

## Additional files

Additional file 1:
**This document provides a description of the WORMSIM model structure, the default parameter quantification as used in the simulations, an annotated input and output files, and instructions on how to run WORMSIM.** (PDF 11191 kb) Additional file 2:
**Zip archive containing the JAVA-based WORMSIM modelling framework (version 2.58Ap9), including example input files (XML) for hookworm transmission and detailed documentation of the XML schema for input files**. The contents of this zip archive are licensed under the Creative Commons Attribution-NonCommercial-NoDerivatives 4.0 International License. To view a copy of this license, visit http://creativecommons.org/licenses/by-nc-nd/4.0/ or send a letter to Creative Commons, PO Box 1866, Mountain View, CA 94042, USA. By opening Additional File X, you agree to the aforementioned license. You are free to use and share (copy and redistribute the material in any medium or format) the material contained within Additional File 4 under the following terms: Attribution — You must give appropriate credit, provide a link to the license, and indicate if changes were made. You may do so in any reasonable manner, but not in any way that suggests the licensor endorses you or your use. NonCommercial — You may not use the material for commercial purposes. NoDerivatives — If you remix, transform, or build upon the material, you may not distribute the modified material. (ZIP 5.69 MB) Additional file 3:
**Methods and results of analyses for sampling error in Kato-Katz slides and the association between mean and aggregation of egg counts in populations.** (PDF 1238 kb) Additional file 4:
**Reproductions of Figs.** **5** **,** **6** **, and** **8** **for alternative assumptions about average and heterogeneity of maximum host egg output (i.e. the level of density dependence in transmission).** (PDF 1530 kb)

## Abbreviations

ALB: Albendazole
Epg: Eggs per gram
MEB: Mebendazole
MDA: Mass drug administration
NB: Negative binomial (distribution)
PC: Preventive chemotherapy
PreSAC: Pre-school-age children
SAC: School-age children
STH: Soil-transmitted helminths
WCBA: Women of childbearing age
WHO: World Health Organization
