# Supplementary material for: Feasibility of controlling hookworm infection through preventive chemotherapy: a simulation study using the individual-based WORMSIM modelling framework
Source: Parasit Vectors. 2015 Oct 22;8:541. doi: 10.1186/s13071-015-1151-4 (PMC4618856; doi:10.1186/s13071-015-1151-4)
Supplement: Additional file 3: — Methods and results of analyses for sampling error in Kato-Katz slides and the association between mean and aggregation of egg counts in populations. (PDF 1238 kb) [file 13071_2015_1151_MOESM3_ESM.pdf]

Additional File 3: Sampling error in Kato-Katz data and the association between mean and aggregation of egg counts

Sampling error in single Kato-Katz slides

To simulate sampling error in hookworm egg counts in WORMSIM as observed in the field, we first used a statistical model to quantify Kato-Katz sampling error, using field data from Uganda [1]. Data consisted of three to four repeated Kato-Katz slides of 41.7 mg, taken over two consecutive days (two slides per day) from 2,037 individuals. We assumed that egg counts from repeated slides follow a negative binomial distribution, parameterized in terms of a mean count and aggregation parameter $k_{\text{Kato-Katz}}$. Individual mean egg counts were assumed to follow a gamma distribution with unknown mean μ_{Uganda} and shape k_{Uganda}. Using Hamiltonian Monte Carlo (HMC) [2], we sampled from the joint posterior distribution of individual mean egg counts, $k_{\text{Kato-Katz}}$, μ_{Uganda}, and k_{Uganda}. Sampling was performed using the No-U-Turn Sampler (NUTS) [3] implemented in the C++ library Stan (version 2.7.0) [4]. The posterior was sampled from using eight parallel Markov chains, each consisting of 500 samples, of which the first half were used for adaptation of the NUTS algorithm and discarded. From the posterior samples, we derived a point estimates (means) and 95%-Bayesian credible intervals (BCI), defined as the 2.5 and 97.5 percentiles of the samples for each parameter.

Average individual egg counts in the Ugandan data were estimated to follow a gamma distribution with mean μ_{Uganda} = 9.45 epg and shape k_{Uganda} = 0.09. Given individual mean egg counts, aggregation of repeated individual egg counts was estimated at $k_{\text{Kato-katz}}$ = 0.40 (95%-Bayesian credible interval: 0.37–0.43; Table A3-1). For simulations in WORMSIM, we used the point-estimate of $k_{\text{Kato-katz}}$ = 0.40.

Endemicity scenarios

For the purpose of predicting the impact of MDA, we defined three endemicity levels representative of field conditions. To do so, we first quantified the association between the average level of infection and the distribution of intensity of infection in a population, based on literature data on the distribution of prevalence of no, light, medium, and heavy infection in different localities [5–11] (data have also been previously described elsewhere [12]). We assumed that in each locality *i*, individual egg counts (underlying the observed prevalence of intensity category) followed a negative binomial distribution with mean μ_i and k_i. To estimate the association between mean and distribution of infection intensity, we assumed that the logarithms of μ_i and k_i follow a bivariate normal distribution with unknown means $(\log(\mu_{\text{Lit}})$

and $\log(k_{\text{Lit}})$) and unknown covariance (V_{Lit}). All parameters (μ_i, k_i, μ_{Lit}, k_{Lit}, and V_{Lit}) were jointly estimated using HMC, as described in the section above.

We found a strong linear association between population-level log-transformed mean egg counts μ_{Lit} and the logarithm of aggregation k_{Lit} (ρ_{Lit} = 0.92, 95%-Bayesian credible interval: 0.78–0.98; Table A3-1). This association seemed to be independent of detection method – McMaster or Kato-Katz (Figure 3 in main manuscript), which may be explained by an earlier finding that these two methods do not perform significantly differently at quantifying of hookworm infection [13] (i.e. they result in similar estimates of mean egg counts). Further, the role of sampling error of the Kato-Katz method is probably relatively small compared to the impact of other sources of heterogeneity in transmission, such as host exposure to infection.

Based on the association between mean and distribution of intensity of infection (parameterized in terms of μ_{Lit}, k_{Lit}, and V_{Lit}), we estimated the value of aggregation parameter k for each endemicity scenario (Figure 3 in main manuscript). Next, we successfully reproduced the expected pre-control distribution of intensity of infection in children aged 10–15 (the typical sample population when investigating infection levels in SAC) in WORMSIM for each endemicity scenario and combination of assumptions about density-dependent worm fecundity (Table A3-2).

References

1. Pullan RL, Kabatereine NB, Quinnell RJ, Brooker S (2010) Spatial and Genetic Epidemiology of Hookworm in a Rural Community in Uganda. *PLoS Negl Trop Dis* **4**: e713.

2. Neal RM (2011) MCMC Using Hamiltonian Dynamics. In: Brooks S, Gelman A, Jones GL, Meng X-L. *Handbook of Markov Chain Monte Carlo*. Chapman and Hall/CRC. pp. 113–162.

3. Hoffman M, Gelman A (2014) The No-U-Turn Sampler: Adaptively Setting Path Lengths in Hamiltonian Monte Carlo. *J Mach Learn Res* **15**: 30.

4. Stan Development Team (2015) Stan: A C++ Library for Probability and Sampling, Version 2.7.0.

5. Montresor A, Urbani C, Camara B, Bha AB, Albonico M, et al. (1997) [Preliminary survey of a school health program implementation in Guinea]. *Med Trop* **57**: 294–298.

6. Albonico M, Bickle Q, Ramsan M, Montresor A, Savioli L, et al. (2003) Efficacy of mebendazole and levamisole alone or in combination against intestinal nematode infections after repeated targeted mebendazole treatment in Zanzibar. *Bull World Heal Organ* **81**: 343–352.

7. Gabrielli AF, Ramsan M, Naumann C, Tsogzolmaa D, Bojang B, et al. (2005) Soil-transmitted helminths and haemoglobin status among Afghan children in World Food Programme assisted schools. *J Helminthol* **79**: 381–384.

8. Phommasack B, Saklokham K, Chanthavisouk C, Nakhonesid-Fish V, Strandgaard H, et al. (2008) Coverage and costs of a school deworming programme in 2007 targeting all primary schools in Lao PDR. *Trans R Soc Trop Med Hyg* **102**: 1201–1206.

9. Pasricha S-R, Caruana SR, Phuc TQ, Casey GJ, Jolley D, et al. (2008) Anemia, iron deficiency, meat consumption, and hookworm infection in women of reproductive age in northwest Vietnam. *Am J Trop Med Hyg* **78**: 375–381.

10. Vercruysse J, Behnke JM, Albonico M, Ame SM, Angebault C, et al. (2011) Assessment of the anthelmintic efficacy of albendazole in school children in seven countries where soil-transmitted helminths are endemic. *PLoS Negl Trop Dis* **5**: e948.

11. Levecke B, Montresor A, Albonico M, Ame SM, Behnke JM, et al. (2014) Assessment of anthelmintic efficacy of mebendazole in school children in six countries where soil-transmitted helminths are endemic. *PLoS Negl Trop Dis* **8**: e3204.

12. Montresor A, À Porta N, Albonico M, Gabrielli AF, Jankovic D, et al. (2015) Soil-transmitted helminthiasis: the relationship between prevalence and classes of intensity of infection. *Trans R Soc Trop Med Hyg* **109**: 262–267.

13. Levecke B, Behnke JM, Ajjampur SSR, Albonico M, Ame SM, et al. (2011) A comparison of the sensitivity and fecal egg counts of the McMaster egg counting and Kato-Katz thick smear methods for soil-transmitted helminths. *PLoS Negl Trop Dis* **5**: e1201.

Tables

Table A3-1. Prior distributions and posterior estimates of Kato-Katz sampling error and association between mean and aggregation of egg counts. Half-normal priors for strictly positive parameters are indicated with a '+' superscript. Log-normal and logit-normal priors are normal distributions for the log and logit-transformations of parameter values, respectively. The first block of parameters (shaded rows) pertain to sampling error in repeated Kato-Katz slides of 41.7 mg. The second block (non-shaded rows) pertains to the association between population mean egg counts and aggregation of eggs counts within populations.

				Posterior estimates		
Parameter	Interpretation	Prior distribution	Prior 95%-BCI	Mean	95%-BCI	Data sources
μ_{Uganda}	Overall mean egg count in Ugandan data	Log-normal$(\log(10), 1)$	1.41–71.0	9.00	7.51–10.74	[1]
k_{Uganda}	Aggregation of individual mean egg counts[a] (shape parameter of gamma distribution)	Normal$^{+}(0, 3)$	0.00–5.88	0.09	0.08–0.10	
$k_{\text{Kato-katz}}$	Aggregation of repeated individual egg counts based on single Kato-katz slides of 41.7 mg[a]	Normal$^{+}(0, 3)$	0.00–5.88	0.40	0.37–0.43	
μ_{Lit}	Overall mean egg count in literature studies	Log-normal$(\log(500), 2)$	9.92–25,198.41	75.79	35.58–146.03	[5–11]
k_{Lit}	Overall mean aggregation of egg counts in literature studies[a]	Log-normal$(\log(0.1), 2)$	0.00–5.04	0.04	0.02–0.06	
$\sigma_{\mu_{\text{Lit}}}$	Between-study standard deviation of logarithm of mean egg count	Normal$^{+}(0, 3)$	0.00–5.88	1.54	1.11–2.15	
$\sigma_{k_{\text{Lit}}}$	Between-study standard deviation of logarithm of aggregation parameter *k*	Normal$^{+}(0, 3)$	0.00–5.88	1.18	0.85–1.62	
ρ_{Lit}	Correlation between study-level mean egg count and aggregation of individual egg counts	Uniform$(-1,1)$	-0.95–0.95	0.92	0.78–0.98	

[a] Lower values for *k* mean more variation in individual egg counts.

Table A3-2. Endemicity scenarios used to predict impact of mass drug administration. WORMSIM transmission parameter values were estimated by means of a grid search for overall transmission rate $\boldsymbol{\zeta}$ and exposure heterogeneity $\boldsymbol{\alpha_{Exi}}$, conditional on $\boldsymbol{k}_{\text{Kato-katz}}$ = 0.40 and assumptions about the saturation level for total host egg output (second column). Figures in bold represent parameter values that were used in the main analysis.

	WORMSIM parameters			Pre-control prevalence of infection in children (age 10–15) as realised in WORMSIM (target values in brackets)				Mean and aggregation of egg counts (target values in brackets)[b]	
Endemicity scenario	Density-dependent worm fecundity in terms of saturation level for host egg output (95%-CI)[a]	Overall transmission rate (ζ)	Exposure heterogeneity (α_{Exi})	Light infection (%)	Medium infection (%)	Heavy infection (%)	Any infection (%)	Mean egg count (epg)	Aggregation parameter (k)
**High**	**1500 (1113–1943)**	**0.7523**	**0.6746**	**73.2 (73.2)**	**8.8 (8.8)**	**6.7 (6.7)**	**88.8 (88.6)**	**1003.9 (1000)**	**0.265 (0.264)**
	1500 (182–4179)	0.7523	0.7558	74.4	8.8	6.2	89.3	959.0	0.274
	2000 (1484–2591)	0.3166	0.8438	72.1	8.7	7.0	87.9	1027.2	0.255
	2000 (242–5572)	0.4089	0.8438	73.1	8.8	6.7	88.6	1001.0	0.264
**Medium**	**1500 (1113–1943)**	**0.1101**	**0.6129**	**52.9 (52.1)**	**2.8 (2.8)**	**1.3 (1.4)**	**57.0 (56.3)**	**289.7 (300)**	**0.107 (0.104)**
	1500 (182–4179)	0.1431	0.4452	53.0	2.9	1.5	57.4	310.5	0.107
	1000 (742–1296)	0.2215	0.3234	51.7	2.8	1.4	55.9	298.3	0.103
	1000 (121–2786)	0.3429	0.2756	51.5	2.8	1.4	55.6	295.6	0.102
	2000 (1484–2591)	0.0924	0.7192	52.7	2.9	1.4	57.0	307.6	0.106
	2000 (242–5572)	0.1009	0.6129	51.3	2.7	1.3	55.3	281.9	0.101
**Low**	**1500 (1113–1943)**	**0.0630**	**0.4467**	**28.0 (27.6)**	**0.9 (0.9)**	**0.3 (0.3)**	**29.3 (28.8)**	**99.7 (100)**	**0.045 (0.044)**
	1500 (182–4179)	0.0630	0.2752	27.7	0.9	0.3	28.9	99.0	0.044
	1000 (742–1296)	0.0721	0.2438	27.8	0.9	0.3	29.1	101.4	0.044
	1000 (121–2786)	0.0825	0.2033	27.4	0.9	0.3	28.6	99.8	0.044
	2000 (1484–2591)	0.0569	0.4746	27.6	0.9	0.3	28.9	101.1	0.044
	2000 (242–5572)	0.0630	0.4467	27.8	0.9	0.3	29.0	98.4	0.044

[a] The 95%-confidence intervals (95%-CI) represent inter-individual heterogeneity in saturation level of host egg output.

[b] These figures were re-estimated from WORMSIM output on prevalence of infection intensity categories, assuming an underlying negative binomial (NB) distribution for egg counts (rather than e.g. directly deriving mean egg counts from individual-level WORMSIM output). This was done for the sake of consistency, as target values for the endemicity scenarios were strictly based on prevalence data [5–11], assuming NB distributions for the underlying distribution of egg counts. However, in WORMSIM (and probably in reality as well), the actual distribution of egg counts within the population does not exactly follow a NB distribution (in WORMSIM, the empirical distribution has a shorter and fatter tail than a NB distribution due to density-dependent worm fecundity), and hence simulated average egg counts are somewhat higher (+30 to 40 epg) than what would be strictly expected under a NB distribution, given some prevalence distribution of intensity categories. The approach we take here avoids this discrepancy, and simply considers the NB distribution a useful metric.
