# Supplementary material for: Feasibility of controlling hookworm infection through preventive chemotherapy: a simulation study using the individual-based WORMSIM modelling framework
Source: Parasit Vectors. 2015 Oct 22;8:541. doi: 10.1186/s13071-015-1151-4 (PMC4618856; doi:10.1186/s13071-015-1151-4)

Additional File 4: Impact of alternative assumptions on predicted trends in infection

This document

Each page of this document features a reproductions of Figure 5, 6, and 8 as found in the main manuscript, but with varying assumptions about the drug used for PC (albendazole *vs.* mebendazole), the average amount of density dependence in worm fecundity (average saturation level in host egg output of 2000 (high) *vs.* 1500 (main analysis), *vs.* 1000 epg (low)), variation between hosts in the amount of density dependence in worm fecundity that worms experience within a host (low *vs.* high inter-individual variation), and larval survival in the environment (average lifespan of 2 *vs.* 4 weeks). For the reader's convenience, we start each set of plots with the plot featured in the main manuscript. Simulated pre-control infection levels may differ somewhat for different plots as for each set of assumptions, WORMSIM transmission parameters were re-tuned based on a grid search.

Figure 5. Impact of targeted preventive chemotherapy with albendazole, as predicted by WORMSIM. The horizontal dashed black indicates the WHO target level of 1% prevalence of medium and heavy infection. Coloured graph lines in each panel represent predicted trends in prevalence of medium and heavy infection in different subpopulations (see legend on the right). Each row of panels represent one of three pre-control endemicity levels, while columns represent different preventive chemotherapy (PC) strategies: annual *vs.* semi-annual and targeting of only pre-school (preSAC) and school-aged children (SAC) *vs.* aforementioned plus women of child bearing age (WCBA). PC coverage is assumed to be 75%, in line with the WHO operational target, and individual participation in PC is determined by a mix of random and systematic factors.

Figure 5-A. Main analysis (albendazole, saturation of host egg output at 1500 epg, low inter-individual variation in saturation, larval lifespan of two weeks).

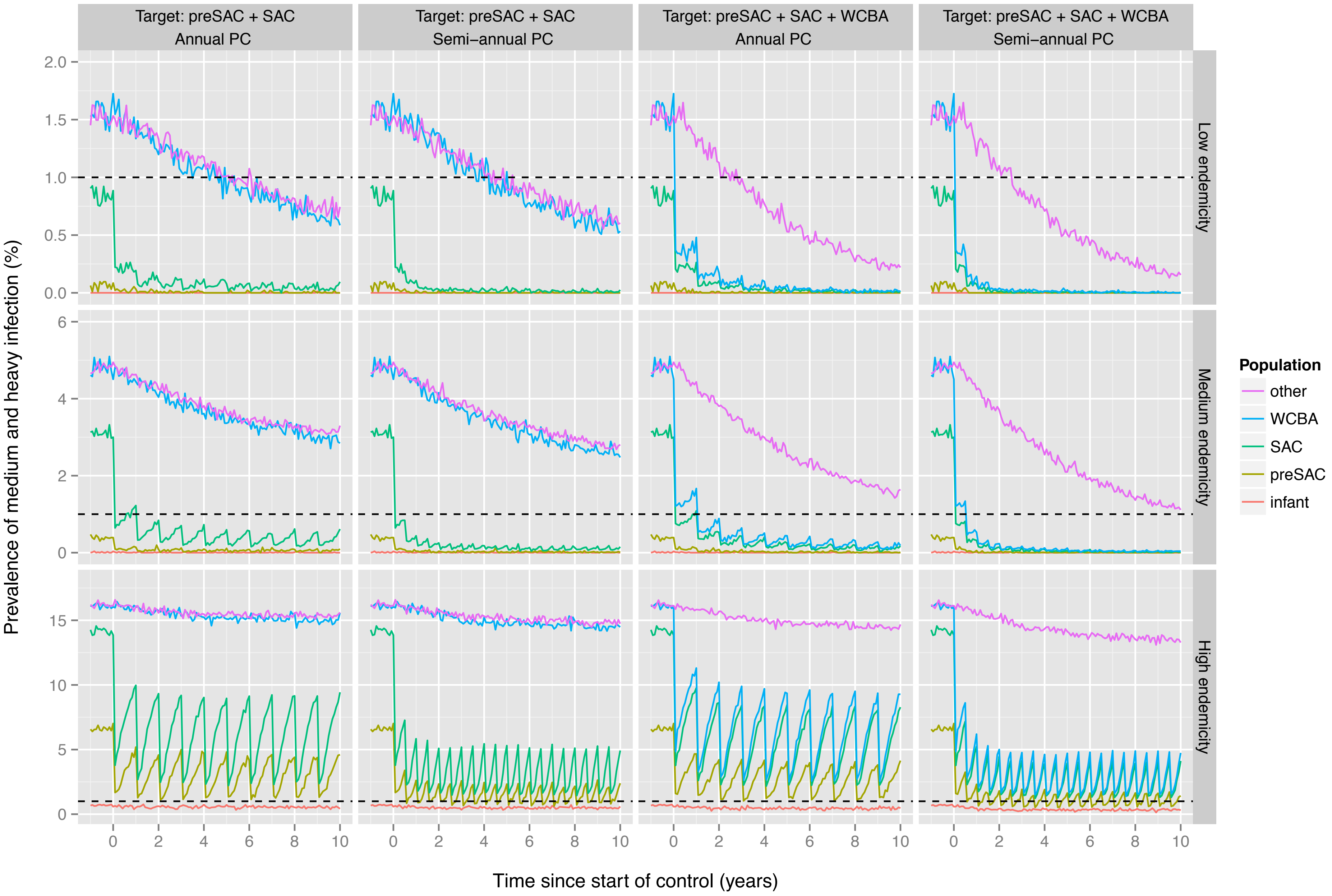

Figure 5-B. Mebendazole.

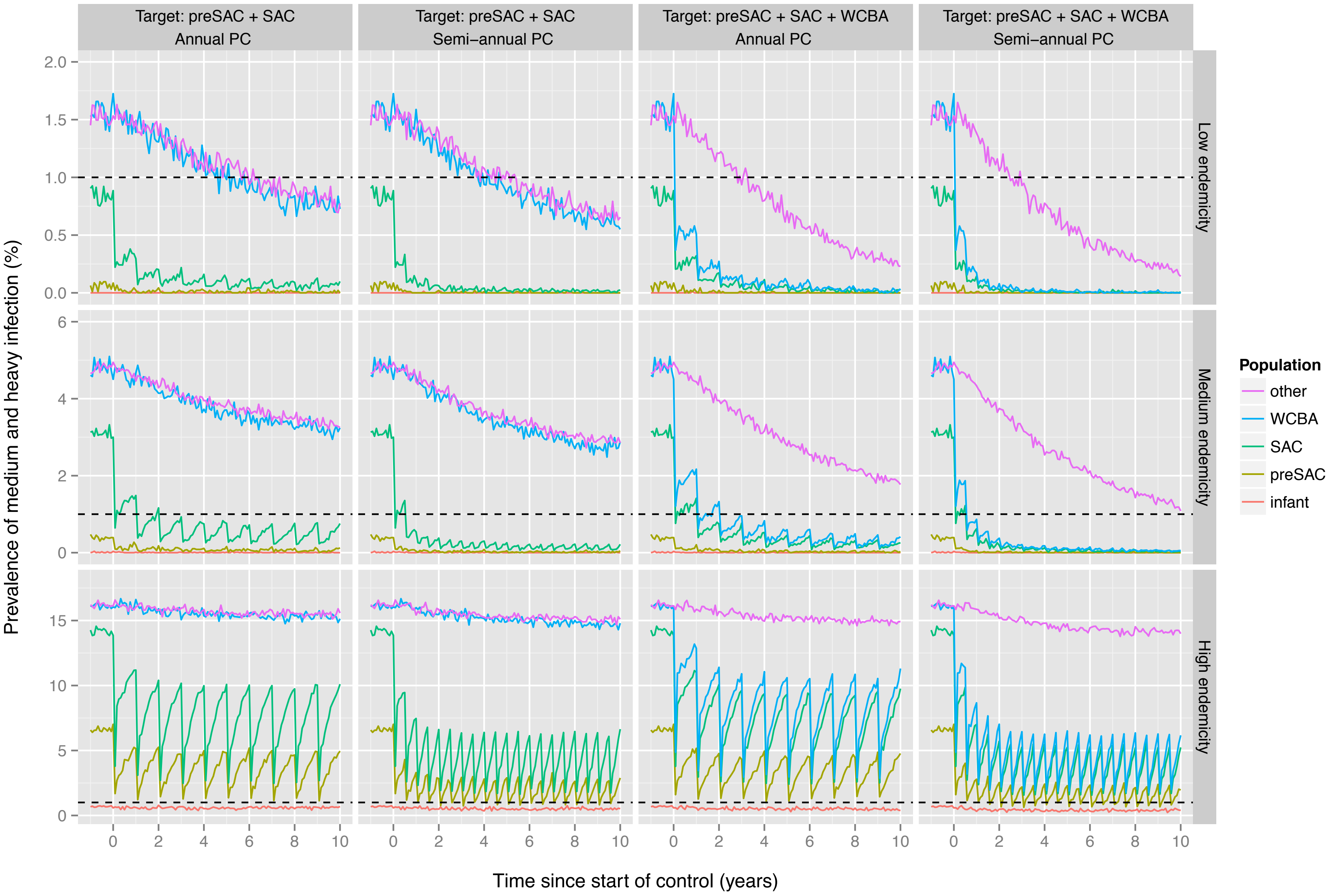

Figure 5-C. Albendazole; higher inter-individual variation in host saturation level for egg output.

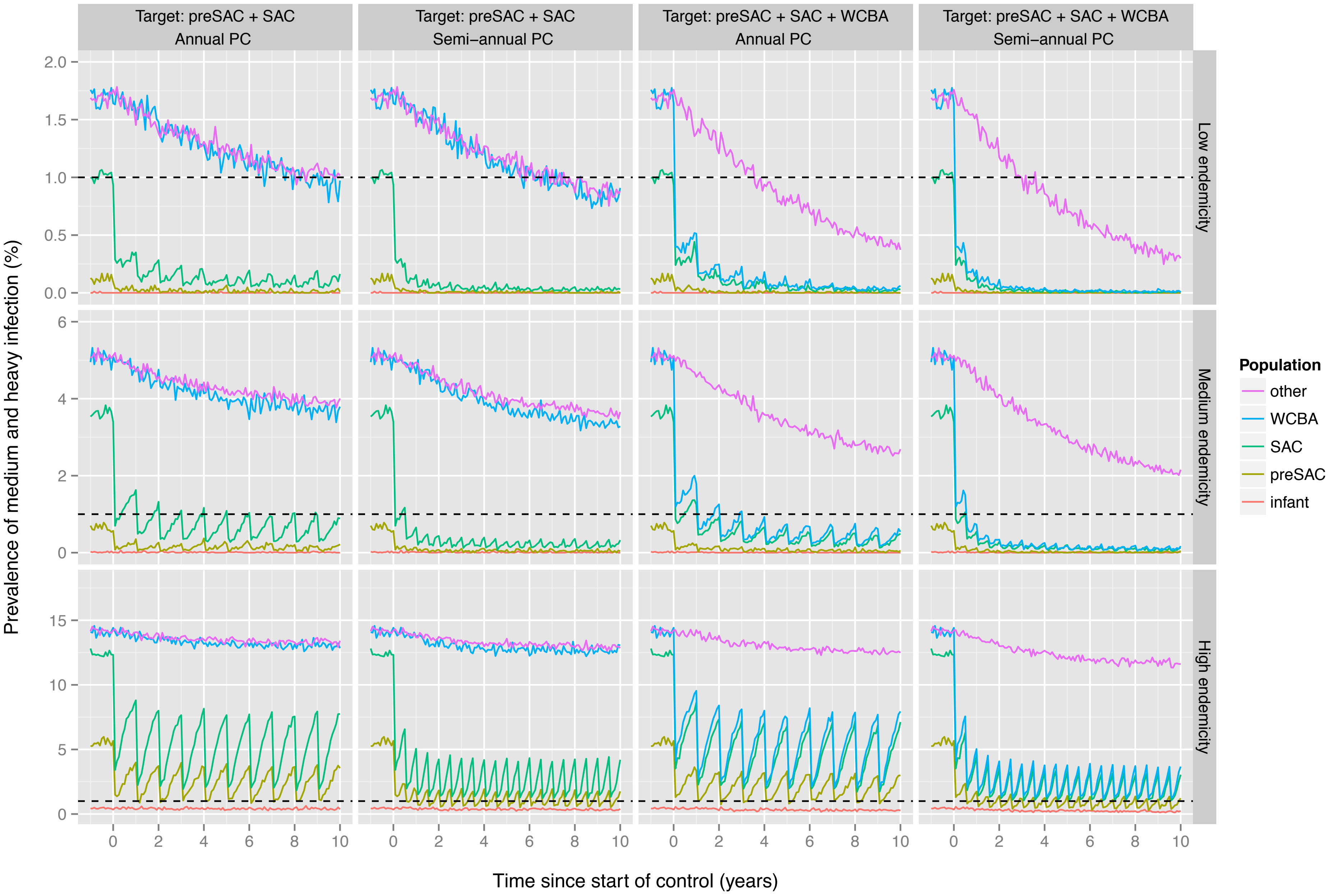

Figure 5-D. Albendazole; higher average host saturation level for egg output.

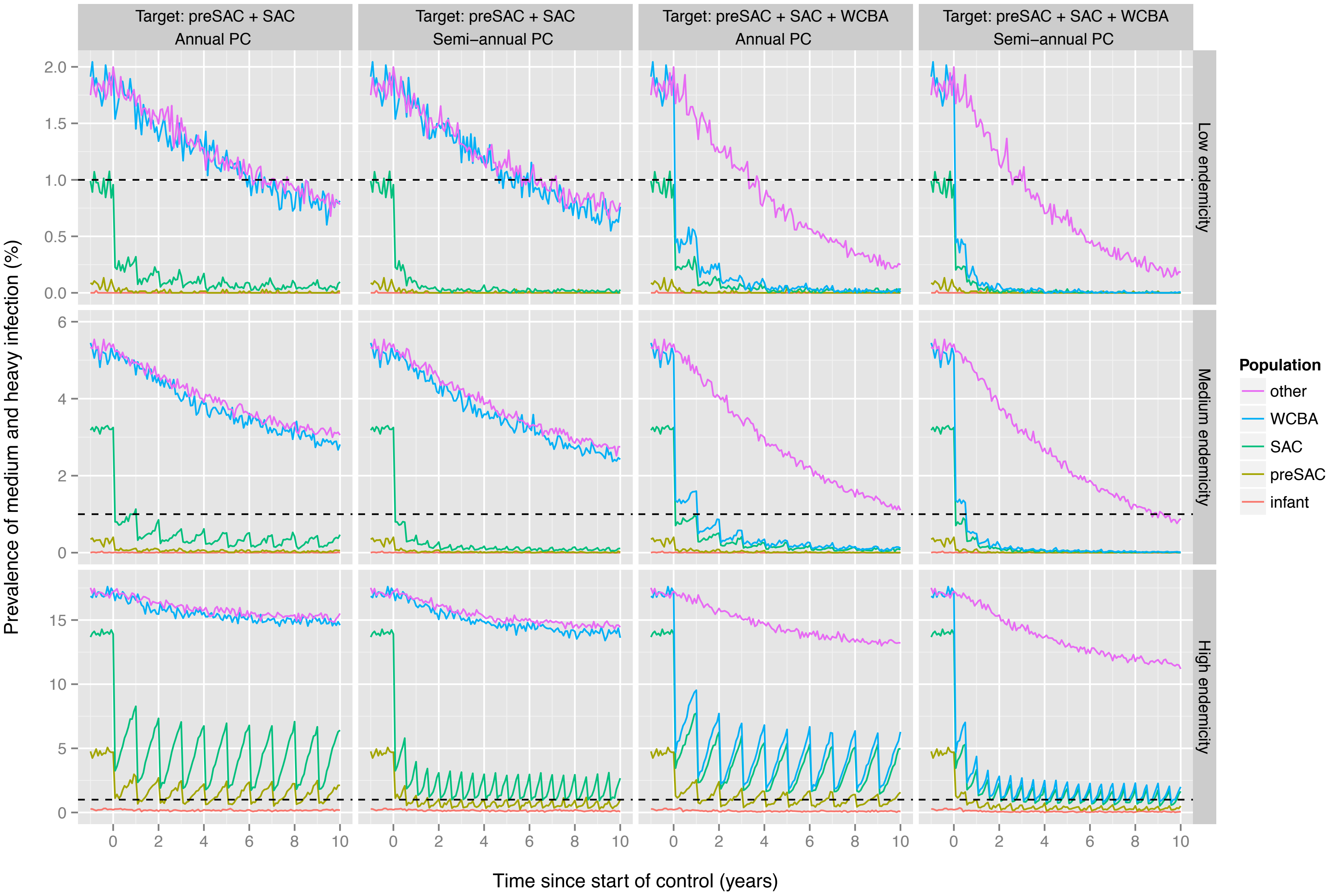

Figure 5-E. Albendazole; higher average and inter-individual variation in host saturation level for egg output.

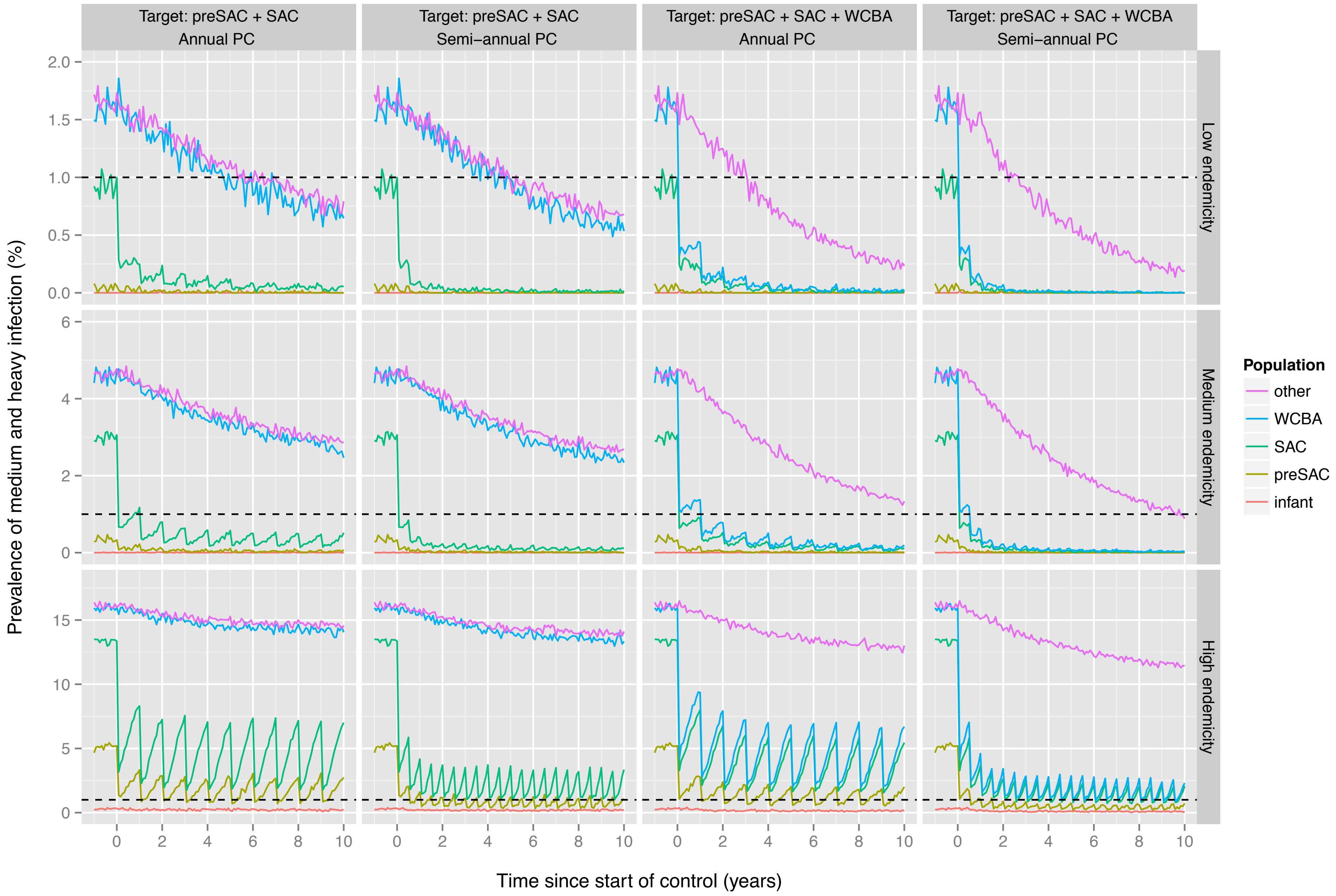

Figure 5-F. Albendazole; higher average larval lifespan in environmental reservoir (four weeks).

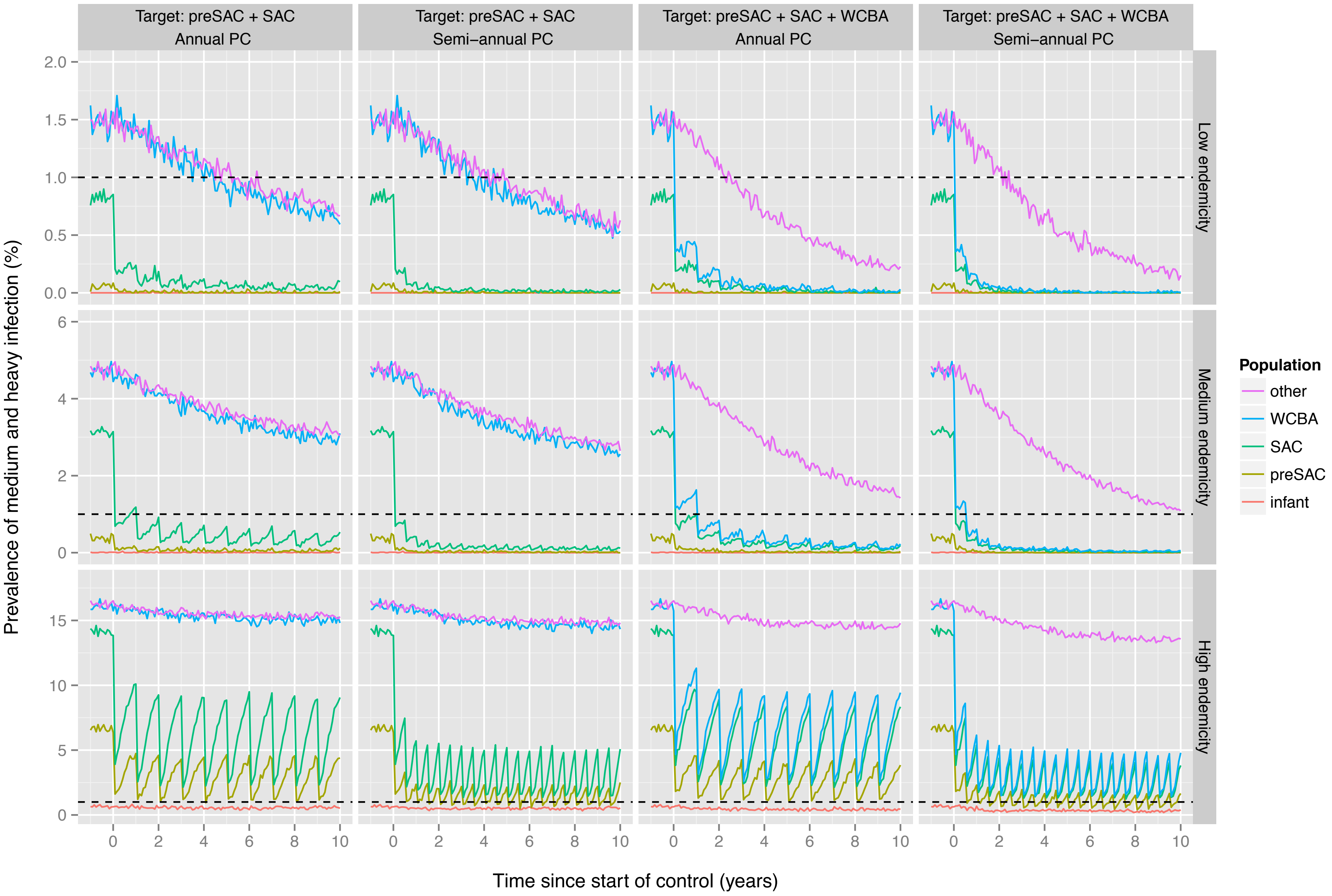

Figure 5-G. Albendazole; lower average host saturation level for egg output.

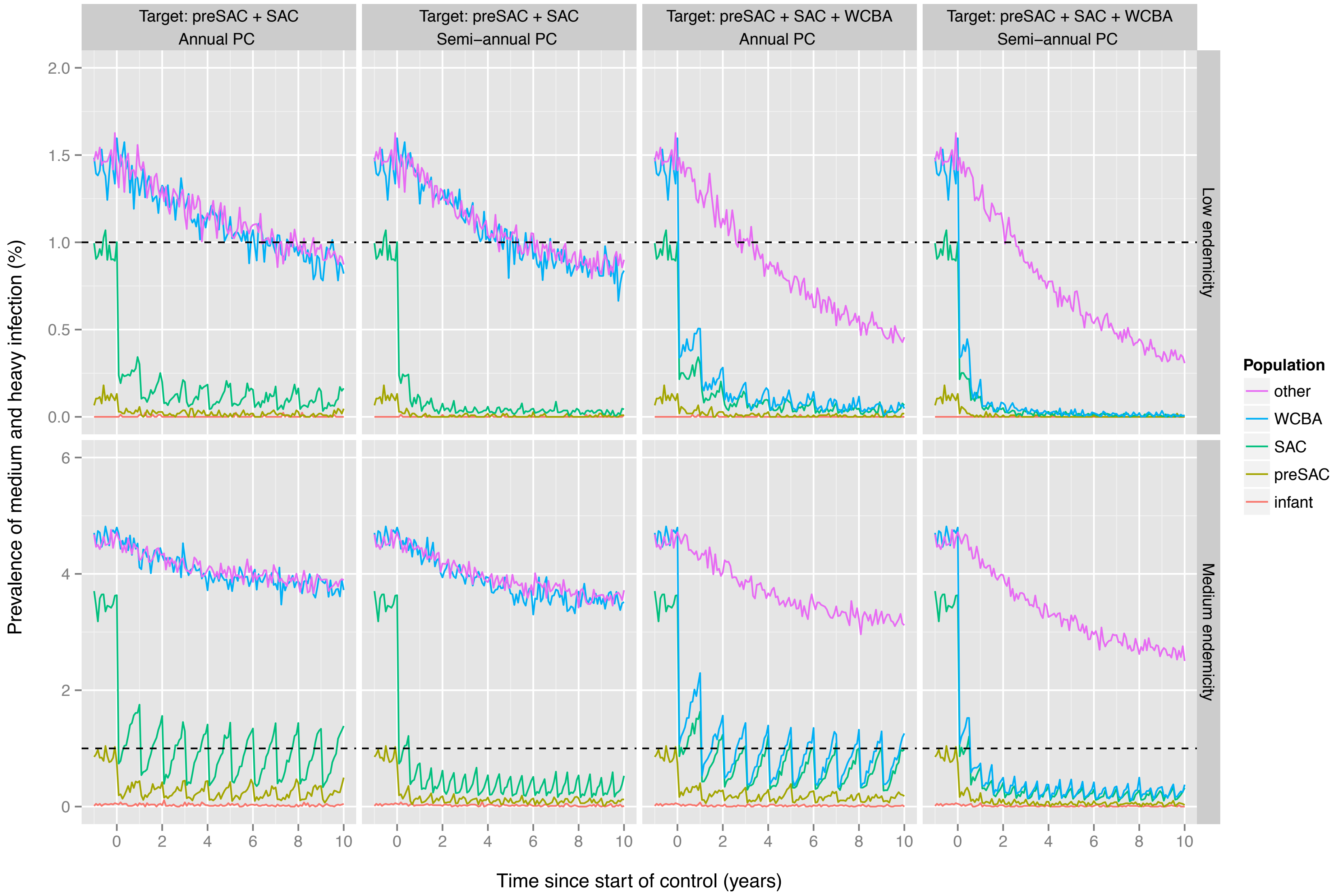

Figure 5-H. Albendazole; lower mean and higher inter-individual variation in host saturation level for egg output.

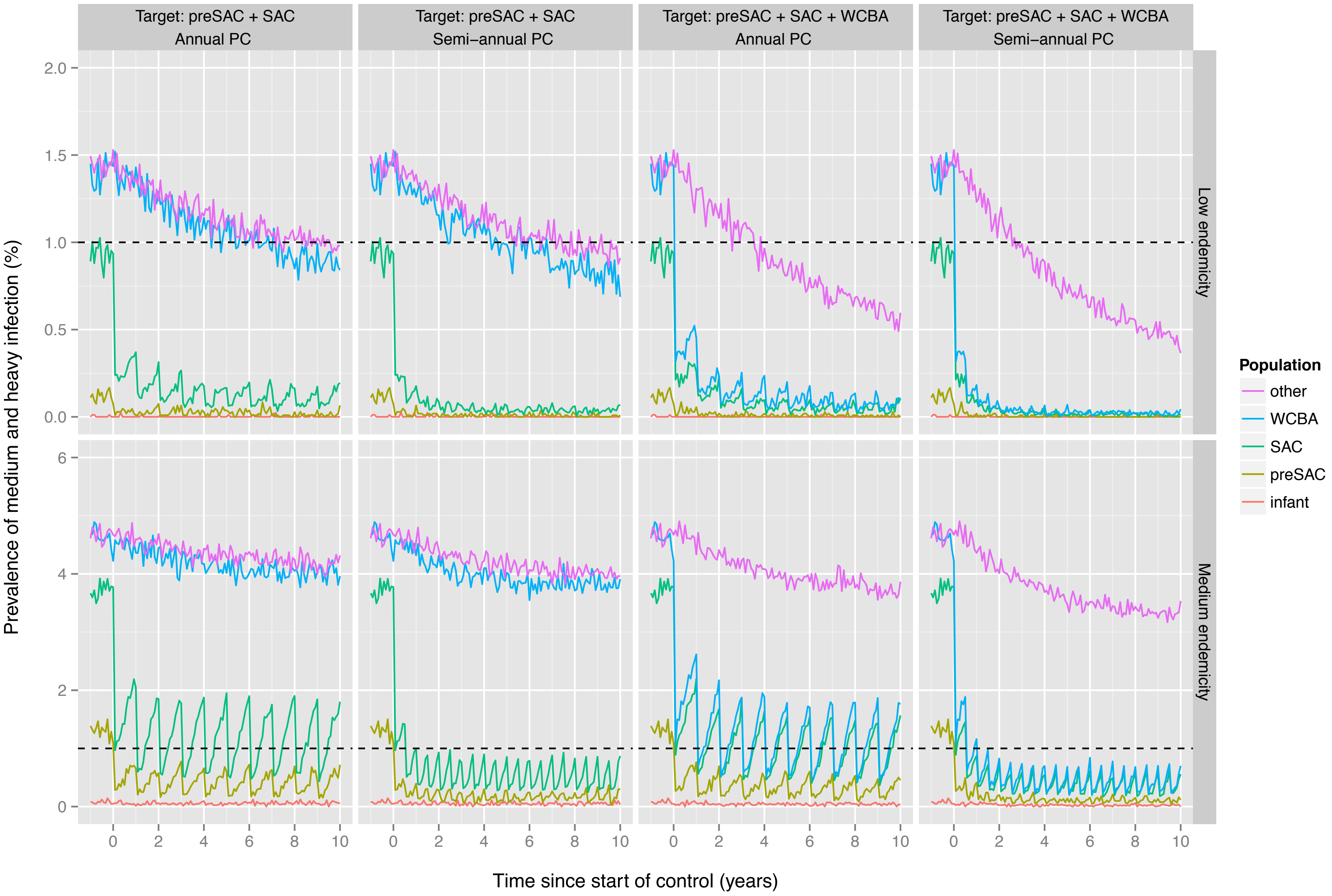

Figure 6. Impact of more intensive preventive chemotherapy with albendazole in highly endemic areas, as predicted by WORMSIM. All panels pertain to the highly endemic scenario. The horizontal dashed black indicates the WHO target level of 1% prevalence of medium and heavy infection. Panels from left to right represent different PC target populations (preSAC, SAC, and WCBA *vs.* total population of age two and above), and PC coverage (75% *vs.* 90%). Panels from top to bottom represent PC implemented at different frequencies (annual *vs.* semi-annual *vs.* four-monthly *vs.* quarterly PC).

Figure 6-A. Main analysis (albendazole, saturation of host egg output at 1500 epg, low inter-individual variation in saturation, larval lifespan of two weeks).

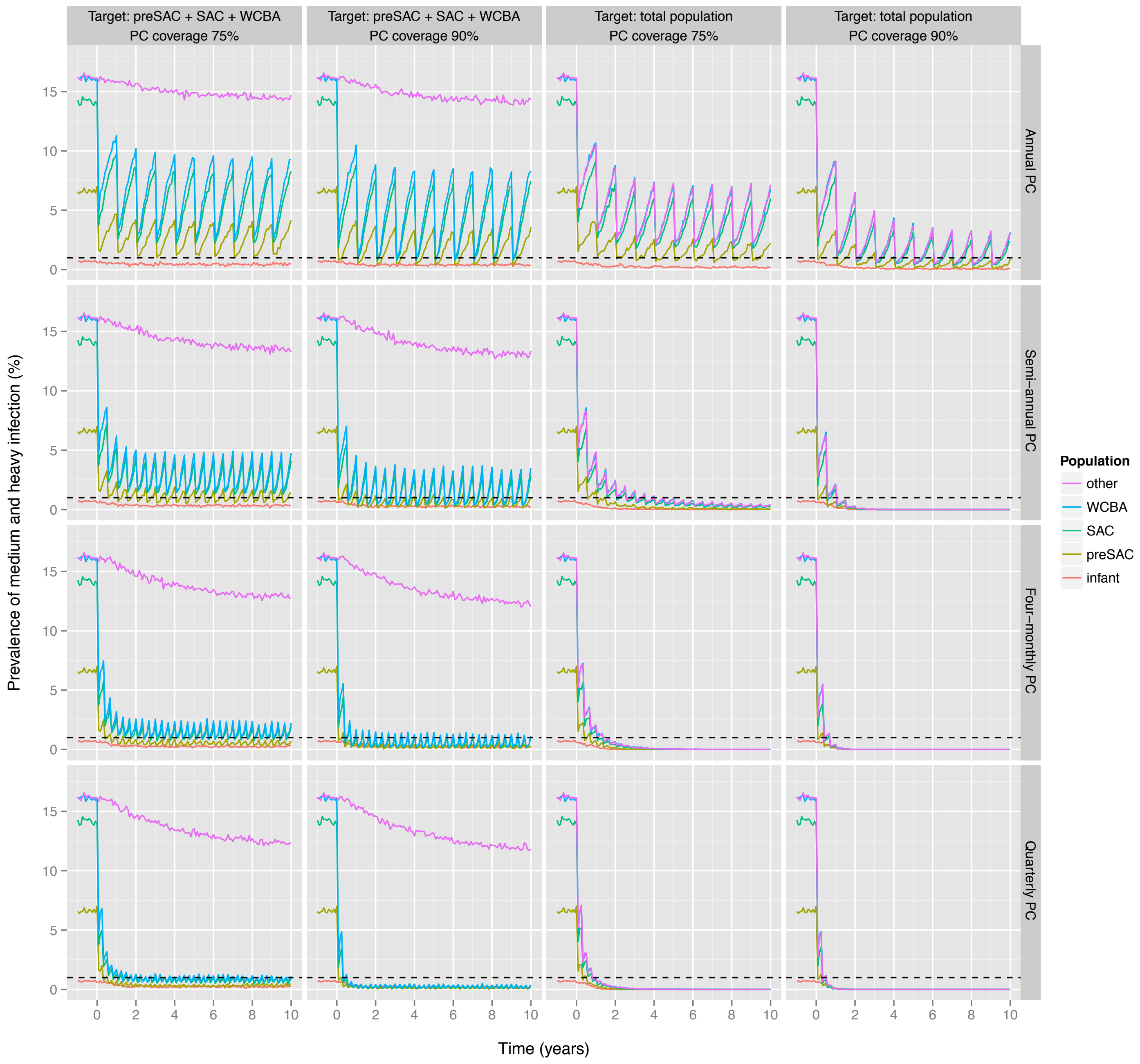

Figure 6-B. Mebendazole.

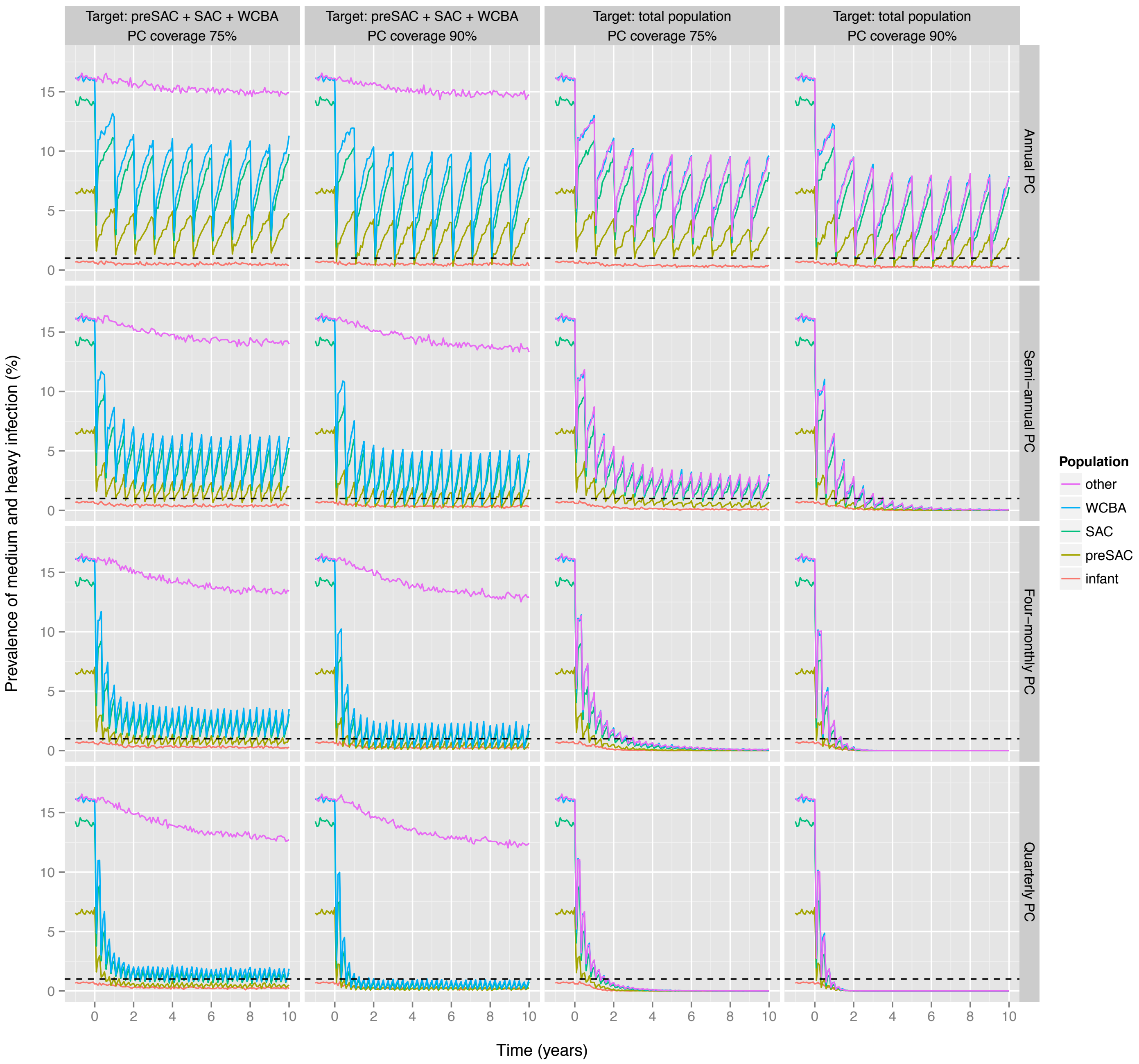

Figure 6-C. Albendazole; higher inter-individual variation in host saturation level for egg output.

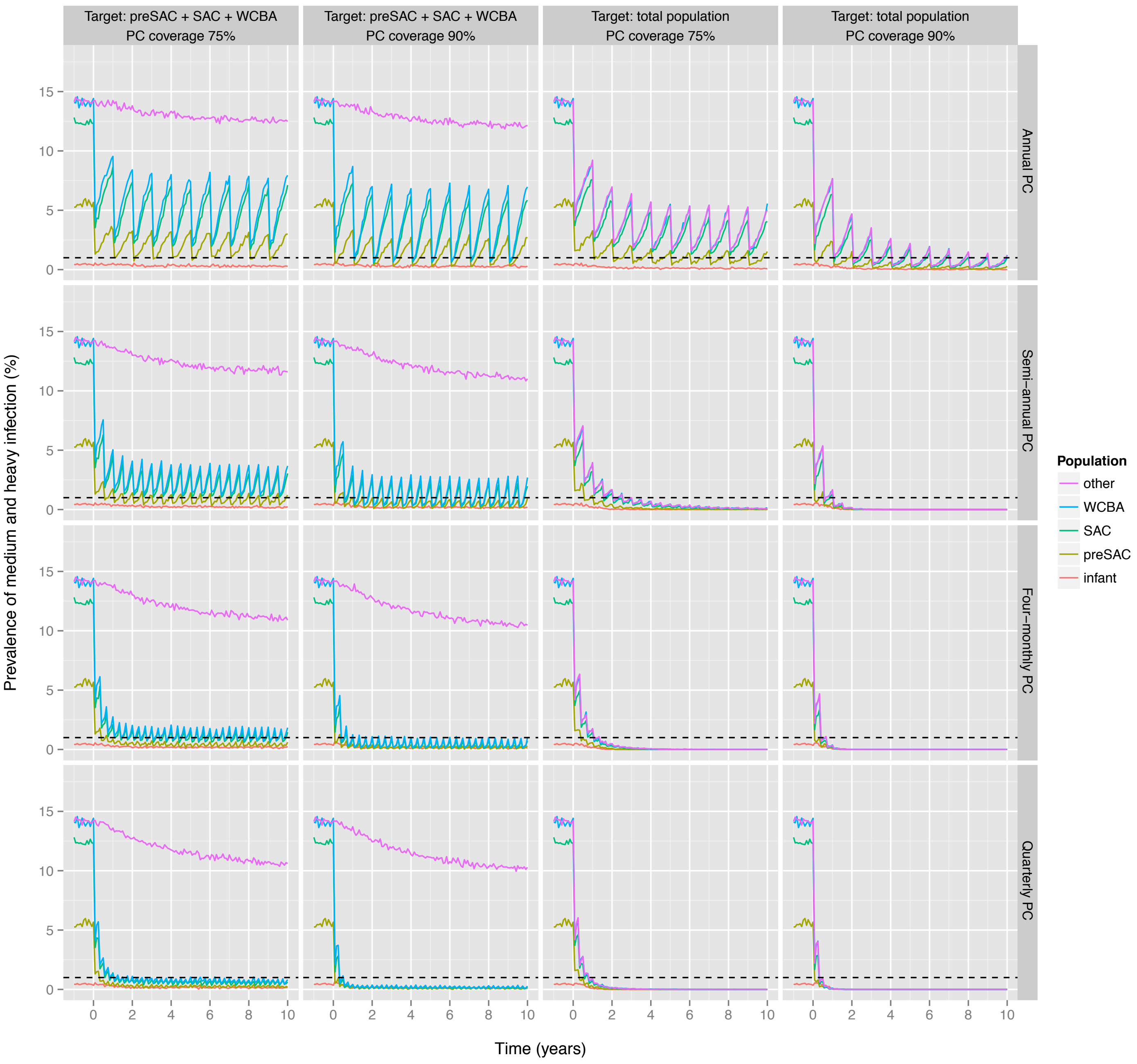

Figure 6-D. Albendazole; higher average host saturation level for egg output.

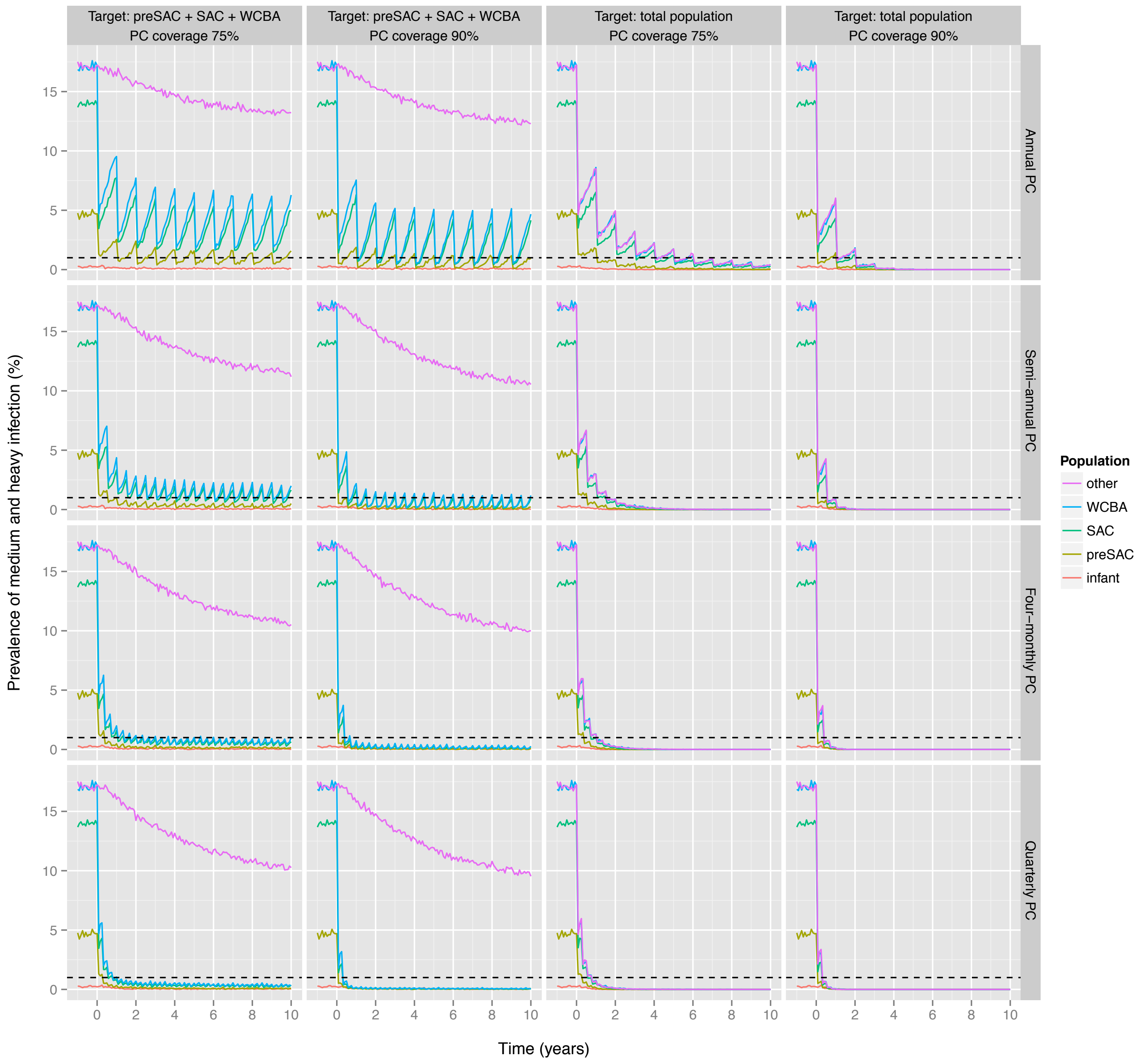

Figure 6-E. Albendazole; higher average and inter-individual variation in host saturation level for egg output.

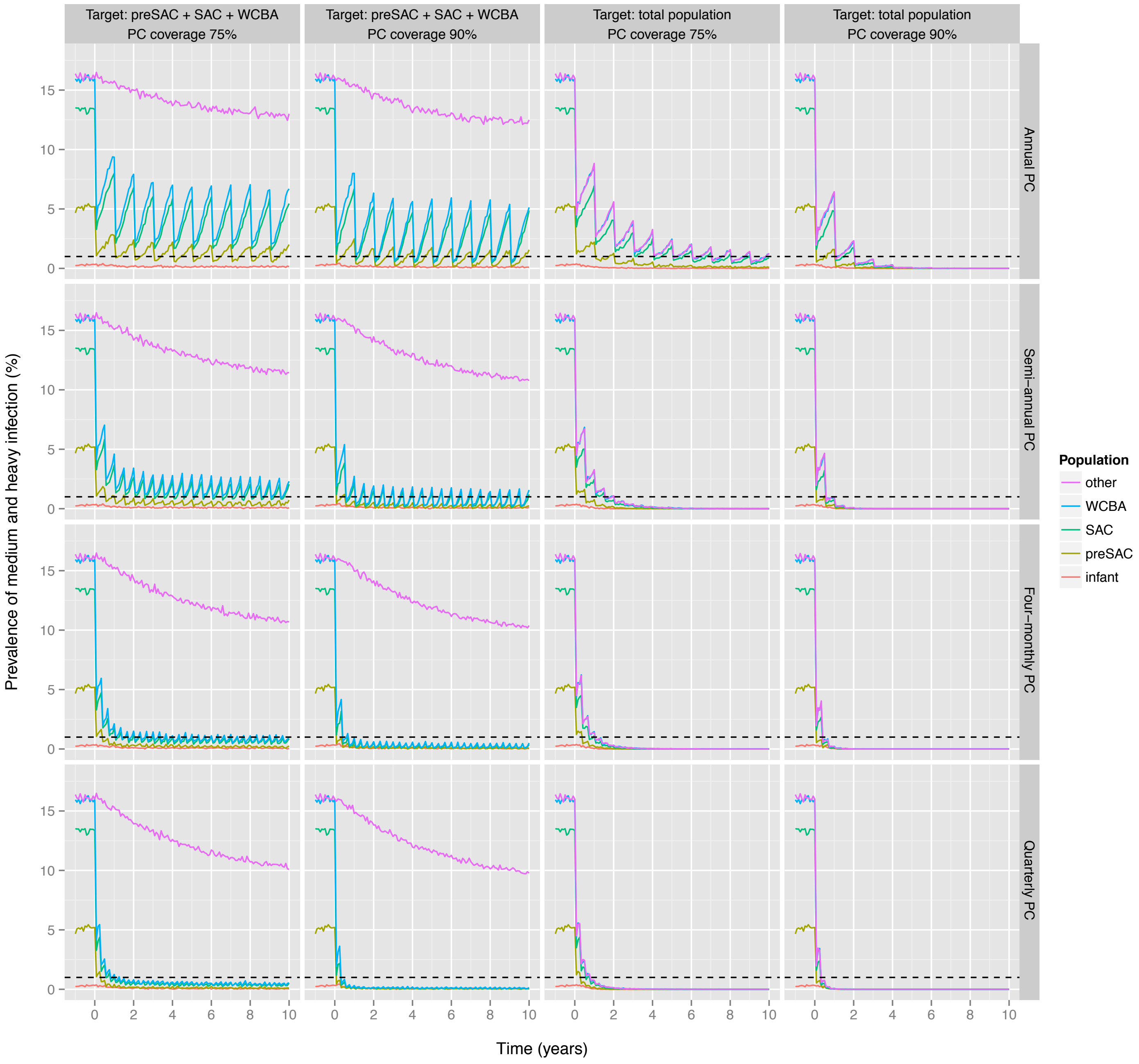

Figure 6-F. Albendazole; higher average larval lifespan in environmental reservoir (four weeks).

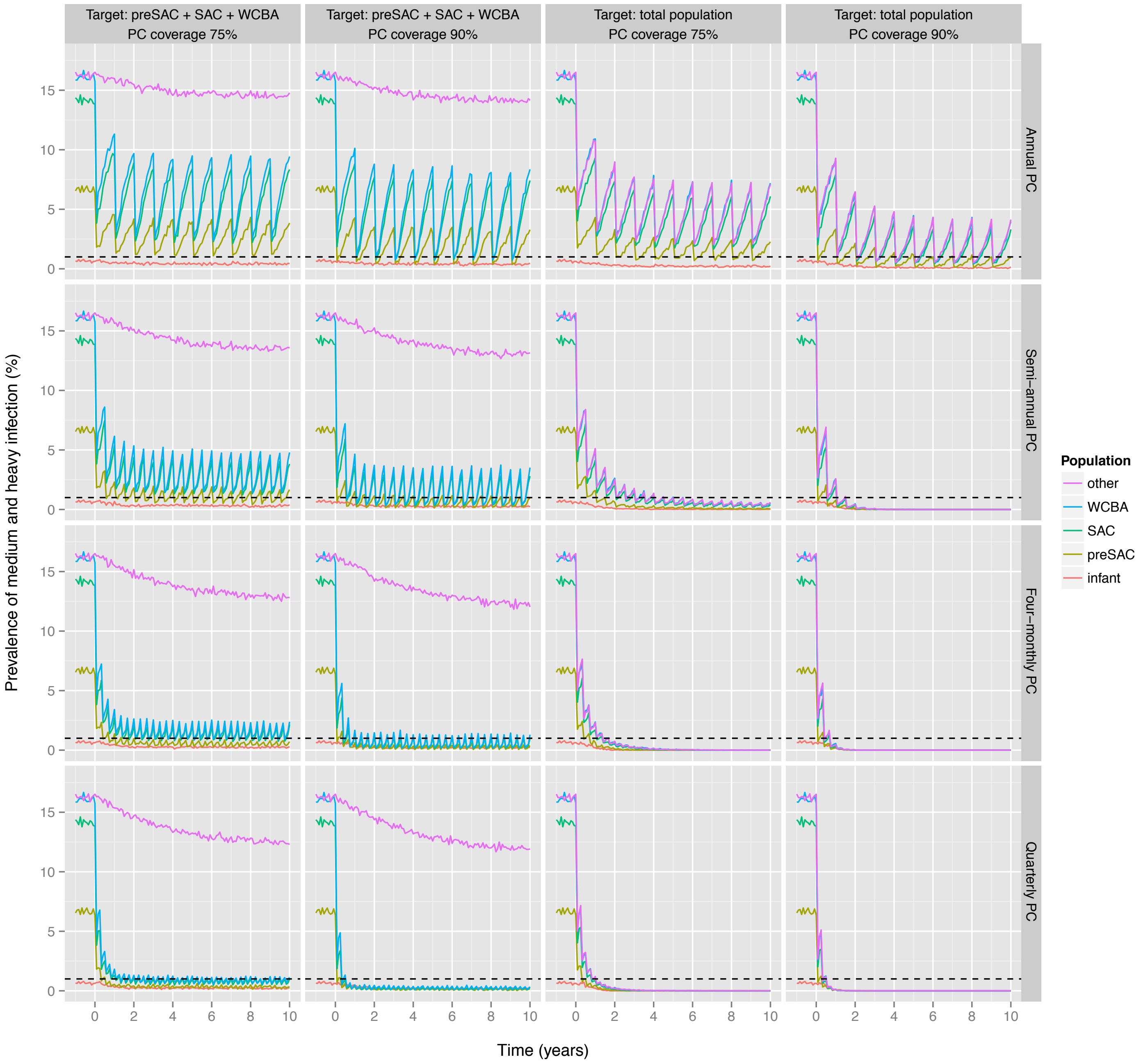

Figure 8. Effect of systematic (non-)participation on impact of preventive chemotherapy with albendazole, as predicted by WORMSIM. All panels pertain to the highly endemic scenario with PC targeted at pre-school and school-age children, and women of childbearing age, implemented at 90% coverage. The horizontal dashed black indicates the WHO target level of 1% prevalence of medium and heavy infection. Panels from left to right represent different patterns in individual participation to PC. Random participation (left column) means that eligible individuals participate completely at random; systematic participation (right column) means that an individual either always participates (if eligible) or never; in the mixed participation pattern (middle column), some individuals are systematically more likely to participate than others (but everyone will participate at some point). Panels from top to bottom represent PC implemented at different frequencies (semi-annual *vs.* four-monthly *vs.* quarterly PC).

Figure 8-A. Main analysis (albendazole, saturation of host egg output at 1500 epg, low inter-individual variation in saturation, larval lifespan of two weeks).

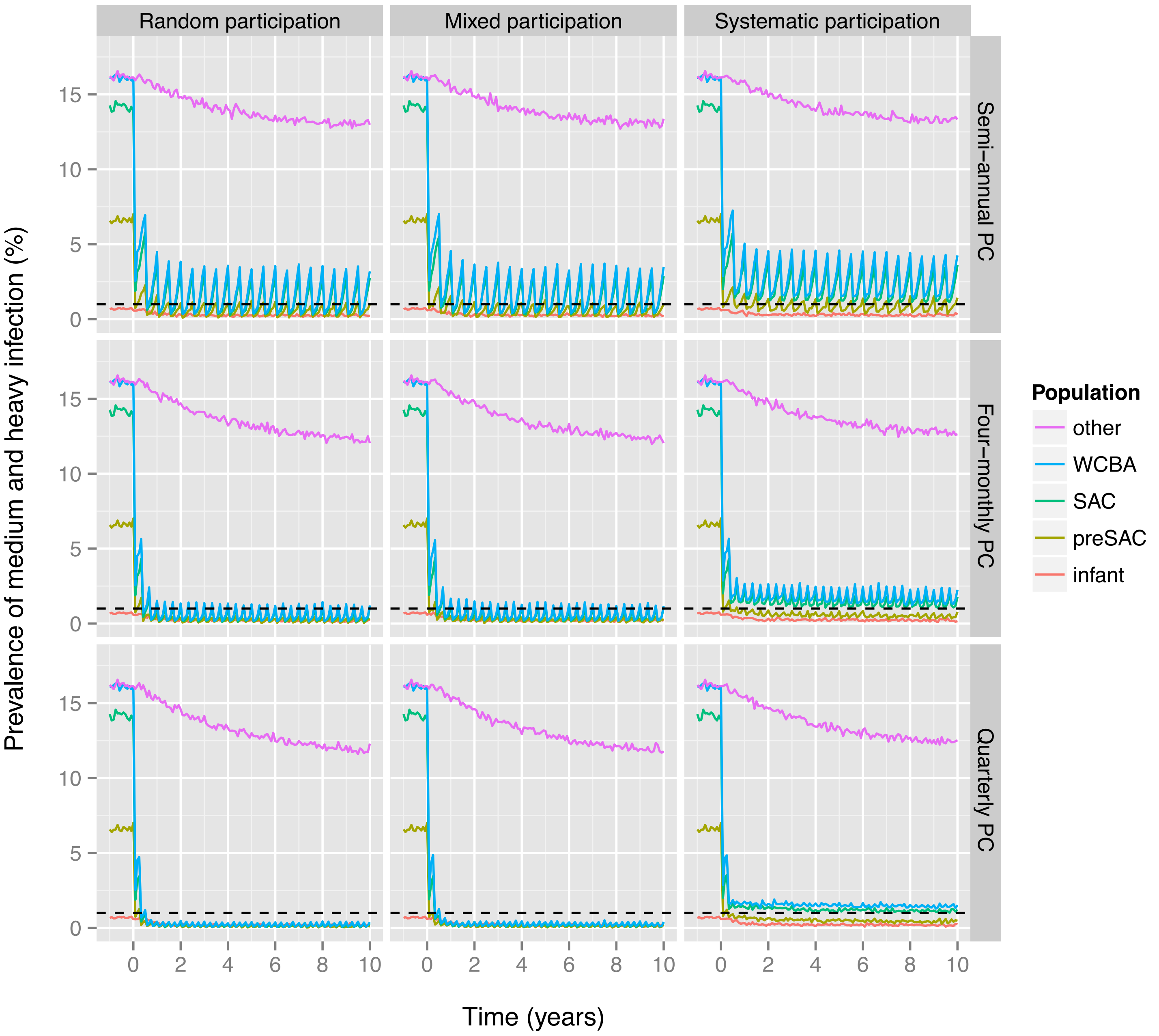

Figure 8-B. Mebendazole.

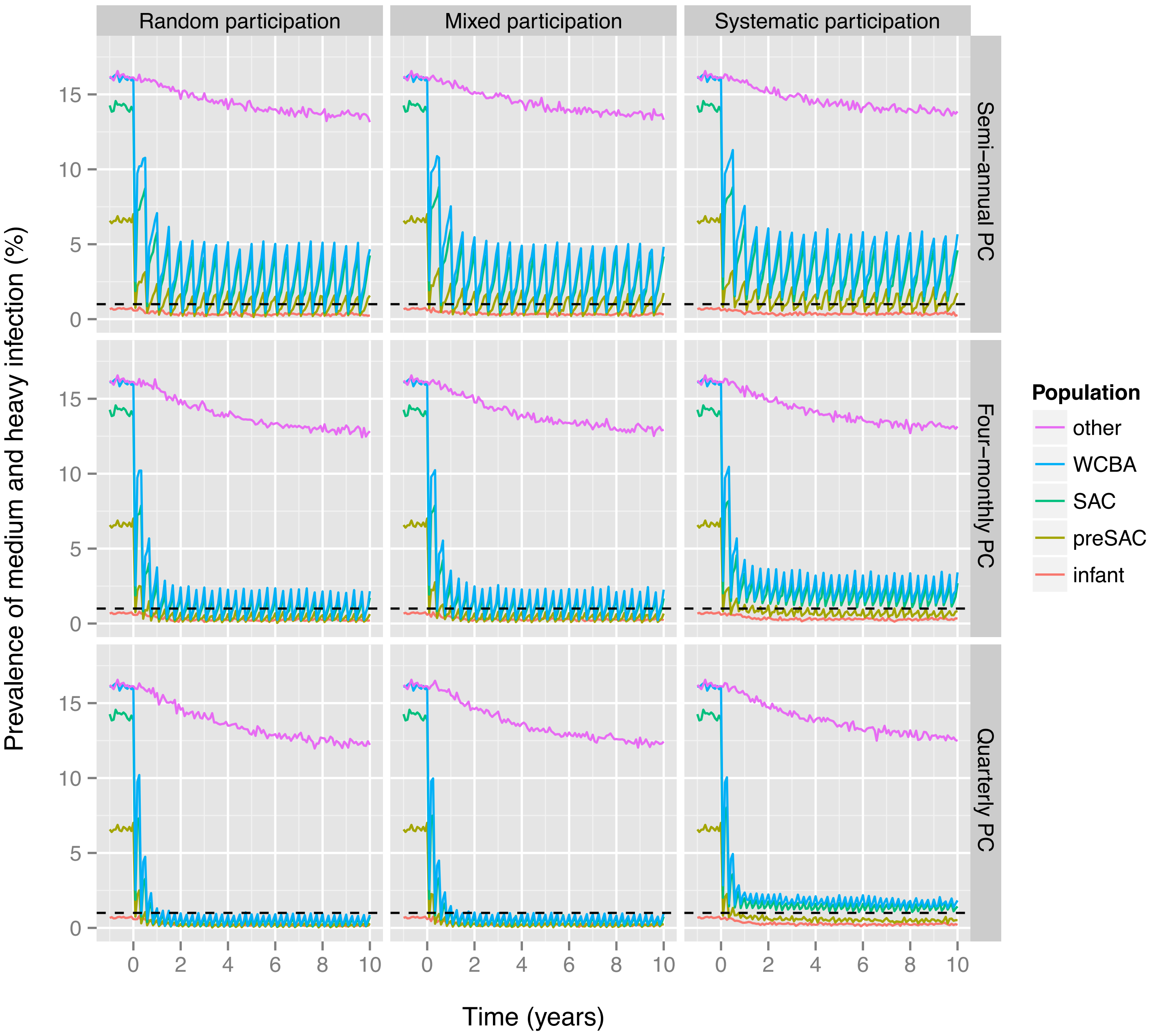

Figure 8-C. Albendazole; higher inter-individual variation in host saturation level for egg output.

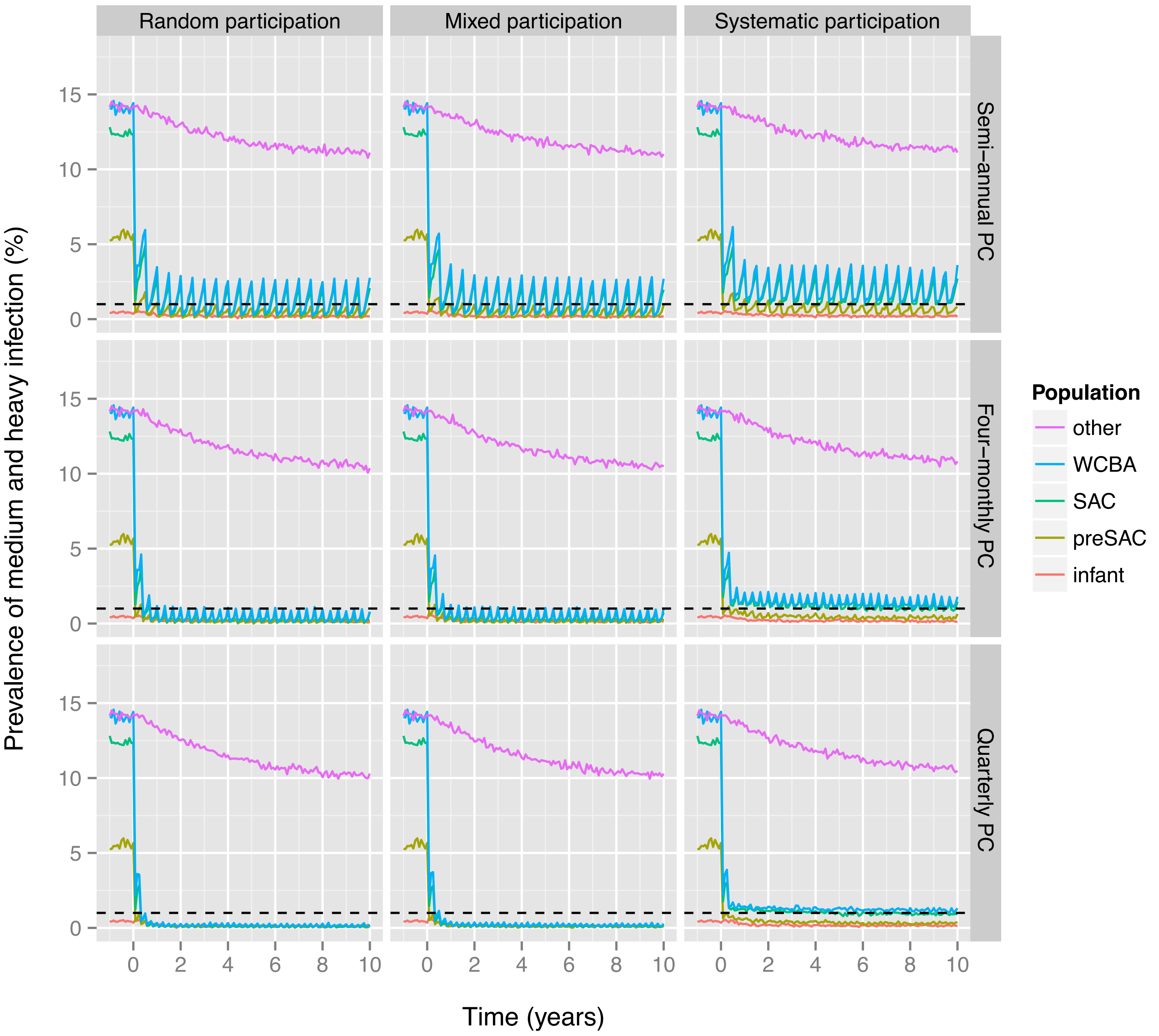

Figure 8-D. Albendazole; higher average host saturation level for egg output.

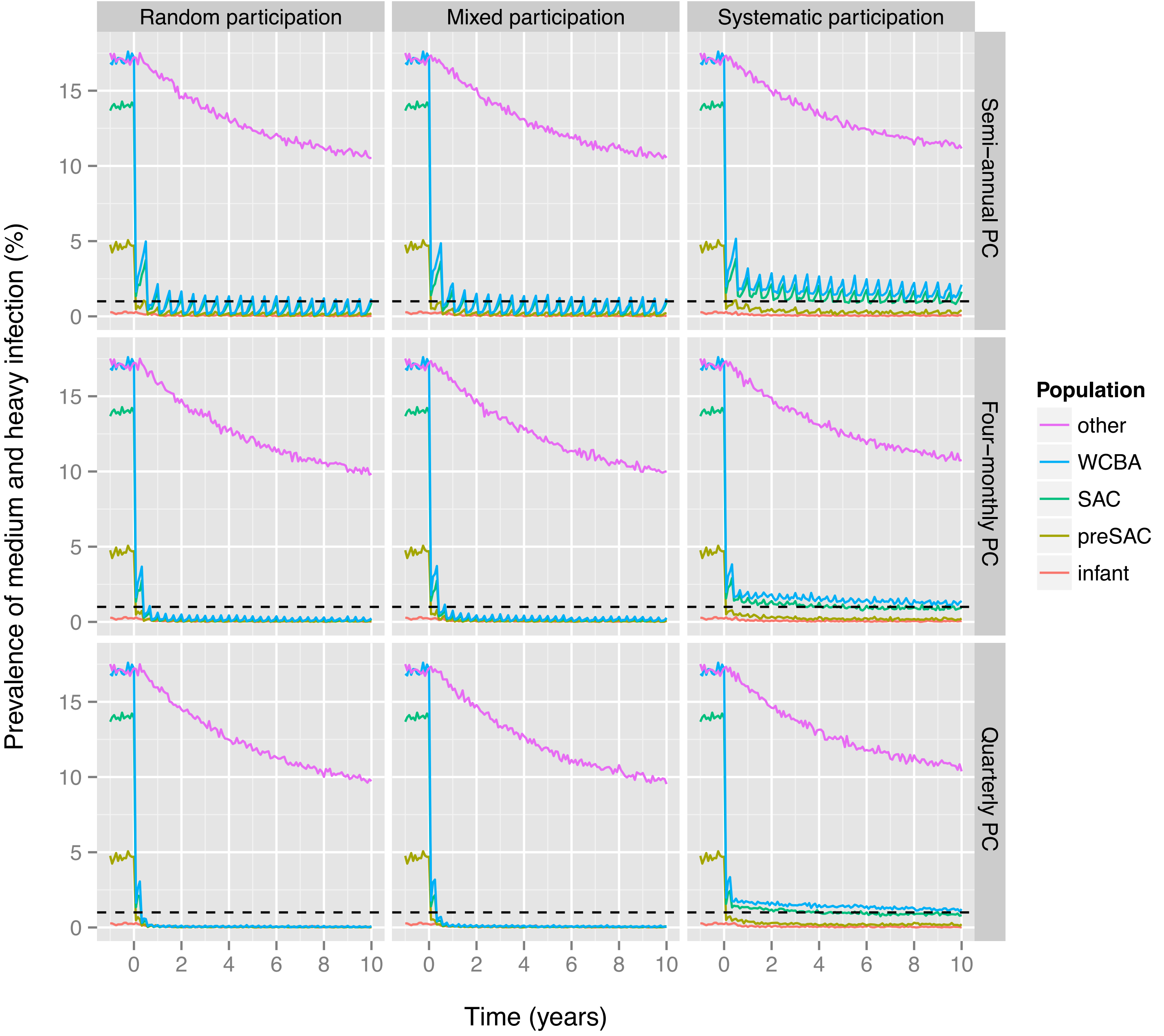

Figure 8-E. Albendazole; higher average and inter-individual variation in host saturation level for egg output.

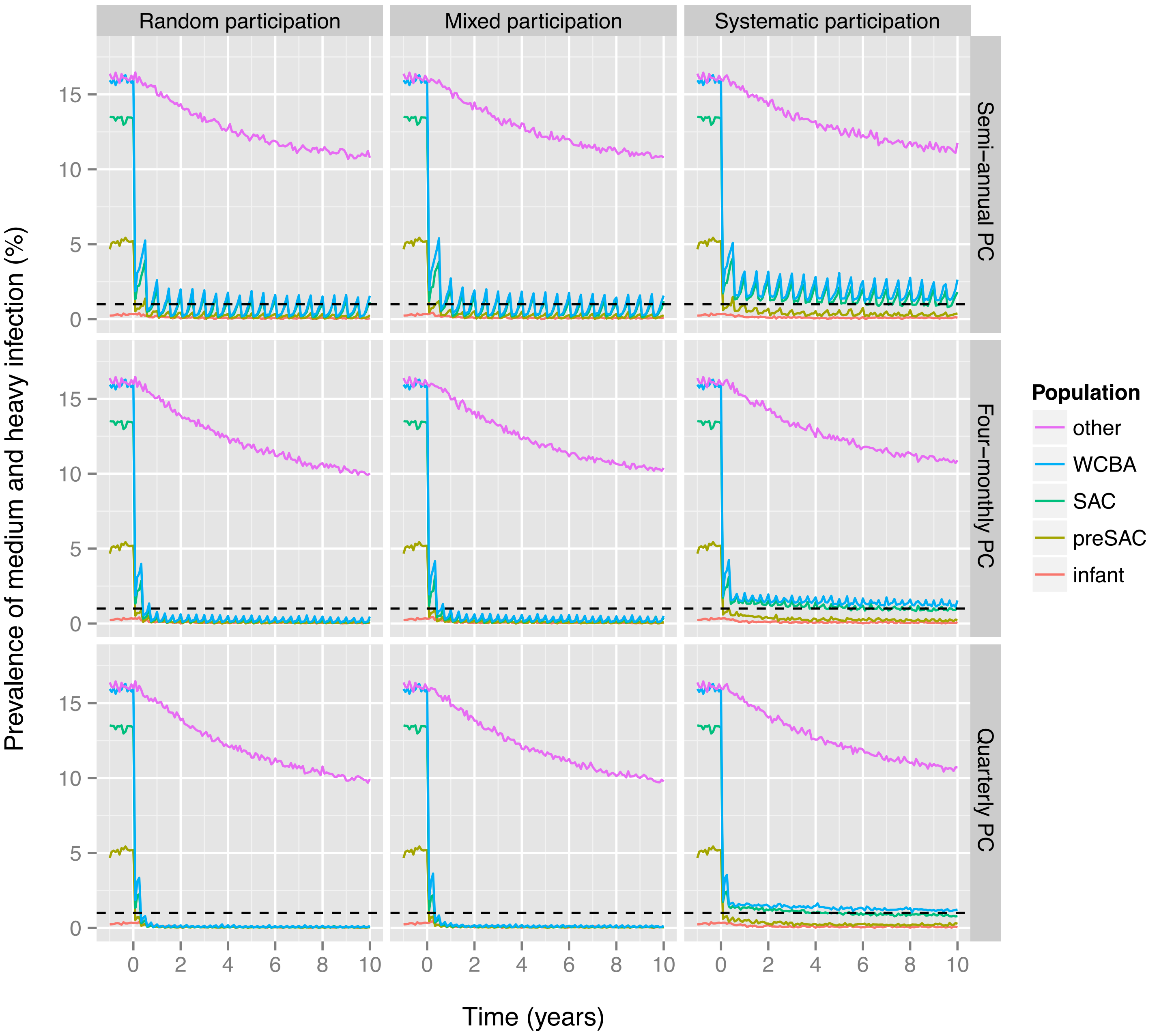

Figure 8-F. Albendazole; higher average larval lifespan in environmental reservoir (four weeks).

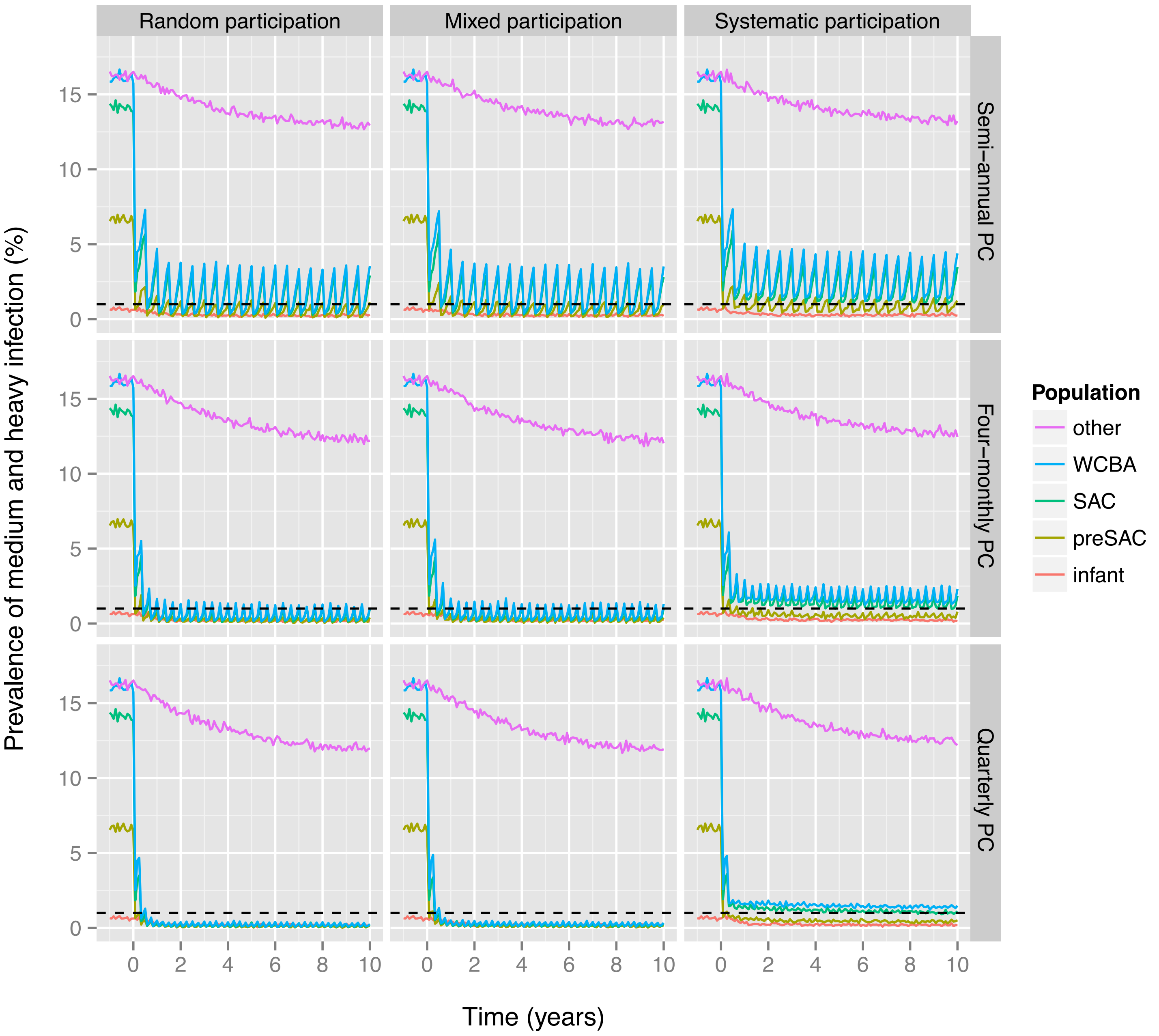

Supplement: Additional file 4: — Reproductions of Figs. 5 , 6 , and 8 for alternative assumptions about average and heterogeneity of maximum host egg output (i.e. the level of density dependence in transmission). (PDF 1530 kb) [file 13071_2015_1151_MOESM4_ESM.pdf]
